# COVID-19 PBMCs are doubly harmful, through LDN-mediated lung epithelial damage and monocytic impaired responsiveness to live *Pseudomonas aeruginosa* exposure

**DOI:** 10.3389/fimmu.2024.1398369

**Published:** 2024-05-21

**Authors:** Clémence Gaudin, Maëlys Born-Bony, Bérengère Villeret, Madeleine Jaillet, Dorothée Faille, Jean-François Timsit, Alexy Tran-Dinh, Philippe Montravers, Bruno Crestani, Ignacio Garcia-Verdugo, Jean-Michel Sallenave

**Affiliations:** ^1^ Laboratoire d’Excellence Inflamex, Institut National de la Santé et de la Recherche Medicale U1152, Physiopathologie et Épidémiologie des Maladies Respiratoires, Université de Paris-Cité, Paris, France; ^2^ Université Paris Cité and Université Sorbonne Paris Nord, Inserm, LVTS, Paris, France; ^3^ Laboratoire d’Hématologie, AP-HP, Hôpital Bichat, Paris, France; ^4^ Réanimation Médicale et des Maladies Infectieuses, Centre Hospitalier Universitaire Bichat-Claude Bernard, Assistance Publique-Hôpitaux de Paris, Paris, France; ^5^ Inserm UMR1148, Laboratory for Vascular Translational Science Bichat Hospital, Paris, France; ^6^ AP-HP Nord, Anesthesiology and Intensive Care Department, Bichat-Claude Bernard University Hospital, Paris, France; ^7^ Service de Pneumologie A, Hôpital Bichat, Assistance Publique des Hôpitaux de Paris, Paris, France

**Keywords:** COVID-19, PBMCs, *Pseudomonas aeruginosa*, neutrophils, proteases, lymphocytes, infection

## Abstract

**Introduction:**

Although many studies have underscored the importance of T cells, phenotypically and functionally, fewer have studied the functions of myeloid cells in COVID disease. In particular, the potential role of myeloid cells such as monocytes and low-density neutrophils (LDNs) in innate responses and particular in the defense against secondary bacterial infections has been much less documented.

**Methods:**

Here, we compared, in a longitudinal study, healthy subjects, idiopathic fibrosis patients, COVID patients who were either hospitalized/moderate (M-) or admitted to ICU (COV-ICU) and patients in ICU hospitalized for other reasons (non-COV-ICU).

**Results:**

We show that COVID patients have an increased proportion of low-density neutrophils (LDNs), which produce high levels of proteases (particularly, NE, MMP-8 and MMP-9) (unlike non-COV-ICU patients), which are partly responsible for causing type II alveolar cell damage in co-culture experiments. In addition, we showed that M- and ICU-COVID monocytes had reduced responsiveness towards further live *Pseudomonas aeruginosa* (PAO1 strain) infection, an important pathogen colonizing COVID patients in ICU, as assessed by an impaired secretion of myeloid cytokines (IL-1, TNF, IL-8,…). By contrast, lymphoid cytokines (in particular type 2/type 3) levels remained high, both basally and post PAO1 infection, as reflected by the unimpaired capacity of T cells to proliferate, when stimulated with anti-CD3/CD28 beads.

**Discussion:**

Overall, our results demonstrate that COVID circulatory T cells have a biased type 2/3 phenotype, unconducive to proper anti-viral responses and that myeloid cells have a dual deleterious phenotype, through their LDN-mediated damaging effect on alveolar cells and their impaired responsiveness (monocyte-mediated) towards bacterial pathogens such as *P. aeruginosa*.

## Introduction

One of the hallmarks of the COVID-19 disease is a decrease in systemic blood circulating T cells, and an increase in neutrophilia, as measured by the N/T ratio. Although a myriad of studies have underscored the importance of T cells, through analyzing their phenotypic and functional characteristics, relatively fewer have studied the functions of myeloid cells in general and neutrophils in particular in COVID disease (1–7). Among neutrophils, low-density neutrophils (LDNs) have been described as having potentially distinct functions. These are cells found in the PBMC fraction of blood samples after isolation from density gradient centrifugation (8–11). They have been sometimes associated with the function of myeloid-derived suppressor cells (MDSCs), which are a heterogeneous mix composed of monocytes (M-MDSCs) and neutrophil (PMN-MDSCs). MDSCs and LDNs have been implicated with a variety of conditions including cancer (12) and auto-immunity, but relatively little is known of their role during infections (12–18). In particular, although studies have described the presence of low-density neutrophils (LDNs) in COVID PBMCs, their role, if any, in the course of the disease remains controversial.

Notably, although some studies have extensively characterized these LDNs phenotypically (surface or granule markers), few studies have investigated their function, and when this was tackled, most experiments aimed at understanding their effect on T cell proliferation. In that context, Cabrera et al. showed in functional assays that COVID LDNs had immunosuppressive capacities (14) and Falck-Jones et al. showed that the levels of blood M-MDSCs in COVID 19 patients were significantly elevated compared to healthy controls and that these cells were suppressive towards allogenic PBMCs (19). Similarly, Schulte-Schrepping et al. demonstrated that severe COVID monocytes were hyporesponsive to LPS *in vitro* (20). In a similar set-up, when total PBMCs were studied, Arunachalam et al. also showed that COVID PBMCs were less responsive *in vitro* towards TLR ligands (21). In addition, Moser et al. showed that whole blood from COVID patients were less responsive to *Candida albicans* lysates (22).

Importantly however, these ‘immunosuppressive’ properties in blood cells may at first sight seem contradictory with the general belief that COVID-19 is characterized by a ‘cytokine storm’ phenotype. However, this conundrum may simply result from the over-activation of immune cells at the lung infective sites, resulting in a tolerized state observed in recirculating peripheral blood cells (23). Indeed, it is well established that neutrophils (albeit mostly normal density neutrophils (NDN) have been studied in that context) can have deleterious effects in lung inflammatory pathologies including acute respiratory distress syndrome (ARDS) (24–26). In that context, it has been shown that roughly 75% of COVID-19 patients admitted in ICU developed ARDS (27) and it is established that alveolar epithelial injury is an important factor in promoting this pathology in ICU patients (28, 29). Among the neutrophilic mediators known to induce lung alveolar injury, proteases are known to be particularly important (24–26), but their role in COVID has not been extensively studied (7, 30–32).

Here, we compared, in a longitudinal study, healthy subjects, idiopathic fibrosis patients, COVID patients who were either hospitalized/moderate (M-) or admitted to ICU (COV-ICU) and patients in ICU hospitalized for other reasons (non-COV-ICU). We show that COVID patients have an increased proportion of low-density neutrophils (LDNs), which produce high levels of proteases (particularly, NE, MMP-8 and MMP-9) (unlike non-COV-ICU patients), which are partly responsible for causing type II alveolar cell damage in co-culture experiments. In addition, we showed that M- and ICU-COVID PBMCs had reduced responsiveness towards further live *Pseudomonas aeruginosa* (PAO1) infection, an important pathogen colonizing COVID patients in ICU (33–36), as assessed by an impaired secretion of myeloid cytokines (IL-1, TNF, IL-8,…). By contrast, lymphoid cytokines (in particular type 2/type 3) levels remained high, both basally and post PAO1 infection, as reflected by the unimpaired capacity of T cells to proliferate, when stimulated with anti-CD3/CD28 beads.

Overall, our results demonstrate that myeloid cells have a dual deleterious phenotype, through their LDN-mediated damaging effect on alveolar cells and their impaired responsiveness (monocyte-mediated) towards bacterial pathogens such as live *P. aeruginosa.*

## Materials and methods

### Subjects

#### Healthy subjects

A group of Healthy volunteers (designated HC/controls, n = 25), who were age (median: 59 [40-68 ] and sex-matched (67% males) with patients from the M-COV and ICU groups (see below) were recruited as part of another study entitled “Evaluation of CD16+ circulating monocyte differentiation into fibrocytes during acute lung injury” (institutional review board ‘CEERB DU GHU Nord’ (No IRN: IRB00006477).

#### Covid patients

Two categories of adult COVID patients were recruited during the spring of 2020 (demographic and clinical data are provided in **Supplementary Table S1**).

a)’Moderate-COVID’ patients (M-COV) were admitted to the Pneumology department of Bichat University Hospital (Paris, France) for a SARS-CoV-2 pneumonia during the spring/summer 2020 (‘first wave’), and agreed to participate in the French COVID cohort study (clinicaltrials.gov NCT04262921; approval of the ethics committee “CPP Ile-de-France VI”, #2020-A00256-33). COVID-19 pneumonia was confirmed by a positive SARS-CoV-2 RT-PCR performed on a naso-pharyngeal swab in all patients. They constituted the Hospitalized patients group (population size of the total cohort: 58, median age 60, 75% males). Altogether, 104 blood samples were obtained: 23 patients gave one sample (on average 12 days after symptoms onset, sample designated M-COV-1), 22 patients gave two samples (on average 12 and 20 days after symptoms onset, samples designated M-COV-1 and M-COV-2, respectively) and 13 patients gave 3 samples (on average 12, 20 days and 3-4 months after symptoms onset, the latter was obtained during a Control visit to the hospital), samples designated M-COV-1, M-COV-2, M-COV-3, respectively).

b)’Severe COVID’ (independent from the M-COV cohort described above) diagnosed for severe pneumonia were transferred to an ICU unit (from Oct 15^th^ 2020 to January 21^st^ 2021) and were subjected to mechanical ventilation or high O2 flow rate therapy (ClinicalTrials.gov Identifier: NCT04344730; EudraCT: 2020-001457-43 (population size of the total cohort: 48, median age 62.5, 69% males). As for M-COV patients, we also obtained, when possible, longitudinal samples: 32 patients gave one sample (on average 4.3 days after ICU admission, samples designated COV-ICU-1), 12 patients gave 2 samples (on average 4 and 11 days after ICU admission, samples designated COV-ICU-1 and COV-ICU-2, respectively) and 4 patients gave 3 samples (on average 4, 11 and 20 days after ICU admission, designated COV-ICU-1, COV-ICU-2, and COV-ICU-3, respectively).

Information on bacterial infection/colonization was obtained on 20 COV-ICU patients. A total of 291 specimens were analyzed. Only the first detection event of any pathogen in a given patient is reported, even when the same pathogen was detected later on, in longitudinal samples. Specifically, bacteria in samples from blood-spoiled catheter, peripheral blood, nose, bronchopulmonary aspiration, tracheal aspiration, bronchial expectoration, bronchoalveolar lavage (BAL), throat, urine, skin, and rectum were obtained (**Supplementary Table S4**). *Candida albicans* and *Pseudomonas aeruginosa* were most prominent, when all body specimens were considered (**Supplementary Table S5**) and, specifically, in respiratory tract samples (**Supplementary Table S6**).

#### Non-COVID ICU ‘acute patients’

A population of non-COVID patients also admitted in ICU (referred as non-COV-ICU, (demographic and clinical data provided in **Supplementary Table S1**) was also investigated in our study (population size of the total cohort: 12, median age 60, 67% males). Patients enrolled were recruited as part of another study entitled “Evaluation of CD16+ circulating monocyte differentiation into fibrocytes during acute lung injury” (institutional review board ‘CEERB DU GHU Nord’ (No IRN: IRB00006477). 6 patients gave one sample (on average 8 days after ICU admission, samples designated non-COV-ICU-1), 2 patients gave 2 samples (on average 8 and 13 days after ICU admission, samples designated non-COV-ICU-1 and non-COV-ICU-2, respectively) and 4 patients gave 3 samples (on average 8, 13 and 22 days after ICU admission, samples designated non-COV-ICU-1, non-COV-ICU-2, non-COV-ICU-3, respectively).

The 12 patients had various pathologies, 6 of them suffering from ARDS or septic shock, and 5 patients were positive for various germs (*Haemophilus Influenzae*, *Streptococcus pneumoniae*, *E. Coli*, *Streptococcus type B*, *Haemophilus Influenzae*, *Staphylococcus aureus*, *Pseudomonas aeruginosa*, *Aspergillus fumigatus*).

#### Patients with idiopathic pulmonary fibrosis

Idiopathic pulmonary fibrosis patients (registered in the European Register of Fibrosis/EuRIPFreg, demographic and clinical data provided in **Supplementary Table S7**, (population size of the total cohort: =17) were, compared to the other populations studied, slightly older (median age: 72 [54-84 ]) and mostly males (82%).

Notably, as shown in **Supplementary Tables S1** and **S7**, there was no major difference in the BMI of the patients, ie: M-COV: 28; COV-ICU: 30, non-COV-ICU: 25; IPF: 29, all in the overweight range.

Notably, IPF patients were sampled in a stable condition, as shown by the very low CRP levels (median of 5, compared to 106, 158, 66.5 for M-COV, COV-ICU, non-COV-ICU patients, see **Supplementary Tables S1**, **S7**).

For all cohorts (HC, M-COV-, COV-ICU, non-COV-ICU, IPF), the actual numbers of samples used are listed in the Figure legends.

### Blood sampling and isolation of PBMCs

All patients routinely underwent a blood test at the time of sampling, including a hemogram and determination of serum parameters (see **Supplementary Table S1**). In addition, for patients and controls, 5 to 20 mls of blood samples were collected in heparinized Cyto-Chex blood collection tubes (Streck, Omaha, NE), stored on ice and then processed within 1-2hrs.

Blood samples were then diluted ½ with PBS and deposited on 20 ml of Ficoll (Lymphocyte Separation Medium, Sigma Aldrich). After 30 minutes of centrifugation at 800g (without brake) at room temperature, the ring of PBMCs was recovered at the interface between the Ficoll and the plasma. The cells recovered were washed in PBS, centrifuged for 10 minutes at 1700 rpm, then resuspended in 1 ml of PBS and counted on a Kova cell after a 1/10 dilution with trypan blue. Finally, 5-10.10 ^ 6 cells per 1 ml of 10% SVF-DMSO were placed in cryopreservation ampoules and stored at -80°C until use.

### PBMC FACS analysis

After washing in complete RPMI medium, PBMCs were first incubated with Fc Block antibody (1/100 dilution, BioLegend) and viability dye (Vital Amcyan au 1/1000, eBioscience) for 15 min at 4°C, washed, and stained with specific antibodies, using both a myeloid mix (CD14 FITC 1/100; CD11b APC/Cy7 1/100; CD15 BV510 1/100; CD16 BV711 1/200; CD66b PE-Cy7 1/100), and a lymphoid mix (CD3 FITC 1/50; CD4 APC/Cy7 1/100; CD8 BV650 1/100; CD33 BV711 1/100; CD19 PE-Cy7 1/100). After incubation 30’ at 4°C in the dark, cells were washed and the pellet resuspended in FACS buffer to perform cytometric analysis with a LSRFortessa cytometer (BD Biosciences). Data were acquired with BD FACSDiva software and analyzed with FlowJo (BD).

### Purification of low-density neutrophils

To isolate low-density neutrophils (LDN) from human PBMC, CD66abce immunomagnetic selection was performed using anti-CD66abce microbeads (Miltenyi Biotec), according to the manufacturer’s instructions.

CD66+ cells (LDN) and CD66- (flow through/FT) cells (containing lymphocytes and monocytes, not shown) were seeded on 96-well plate at 200,000 cells/well in serum-free RPMI 1640 medium and cultured for 4 h. Supernatants were collected for protein (ELISA) and enzymatic activities analysis.

Independently, in a subset of experiments, LDNs were incubated with labelled allogenic PBMCs or with labelled syngeneic FT cells containing T cells. T lymphocyte proliferation was assessed as described below.

### T Lymphocyte proliferation and FACS analysis

PBMCs or ‘flow-through cells’ (FT, see above) were labelled with Cell Trace Violet (2µM per 10^6^ cells/ml, 10 min at 37°C). After centrifugation 10 min at 1700 rpm, PBMCs or FT cells (+/- LDN, see above) were resuspended in R20 medium (RPMI 1640 medium supplemented with 20% FBS, 1% Pen/Strep, 50µM β-mercaptoethanol, 1X sodium pyruvate, 0,1% gentamycin, 1X non-essential aminic acid) and *CTS* OpTmizer *medium, respectively (*200,000 to 400,000 cells per well). Cells were then stimulated with 20ng/ml rIL-2 (PeproTech), plus anti-CD3 and anti-CD28 Dynabeads (average of 1 per cell, Gibco) in a final volume of 150 ul/well, and according to the manufacturer’s instructions. After 4 days incubation at 37°C in a 5% CO2 atmosphere, cells were retrieved, washed in FACS tubes, blocked as above, washed and labelled with an antibody mix of CD3 FITC 1/50, CD4 BV650 1/50 et CD8 APC/Cy7 1/50. After incubation and FACS acquisition, as detailed above, lymphocyte proliferation was assessed by Cell Trace Violet (CTV) dilution.

### A549 cells – PBMC co-culture

A549 cells (ATCC number CRM-CCL-185) were seeded in 24-well plate at 150 000 cells/well in 0,5 mL of F12K medium supplemented with 10% FBS and 1% penicillin-streptomycin and cultured for 24 h (37°C, 5% CO_2_). When sub-confluent, they were co-cultured with 5x10^5^ PBMCs in serum-free DMEM supplemented with 1% Pen/Strep, 0,1% gentamycin and seeded on A549 cells for overnight incubation (37°C, 5% CO_2_). Supernatants were collected for protein (ELISA) and enzymatic (NE, MMP) activities. Epithelial cell damage was assessed, using the Fiji ImageJ software, supernatants were collected for LDH protein content, and measurement of enzymatic activities (NE, MMP) was performed.

### PBMCs infection with PAO1

PBMCs, LDN or FT cells from control HC and COVID patients were washed in Dulbecco′s Modified Eagle′s Medium (DMEM) and centrifuged for 10 minutes at 1700 rpm. After counting with trypan blue on Kova cells, 2.10^6 cells in a 24-well cell culture plate (Corning™3524) were either mock-stimulated, or infected with Pseudomonas aeruginosa (PAO1, ATC 15692), as described in ref (37), at a multiplicity of infection (MOI) of 1, in serum-free DMEM medium. After for 4hrs at 37°C, PBMCs were recovered, centrifuged for 10 minutes at 1700rpm, and the supernatants were collected for further analysis.

### Enzymatic activities

All enzymatic activities were performed in 384 black wells plates in a final volume of 30 µL, using the procedure described in ref (38).

### ELISAs

IL-1b, TNF-a, IL-8, IFN-g, IL-13, IL-17 ELISAs were all DuoSet, from R&D (catalog numbers respectively DY201, DY210, DY208, DY285B, DY213, DY317).

### Luminex analysis

PBMCs from healthy controls (HC, n= 25), and from IPF (n= 16), M-COV-1 (n= 19), M-COV-2 (n= 16), M-COV-3 (n= 7), COV-ICU-1 (n= 6), COV-ICU-2 (n= 6), COV-ICU-3 (n= 2), and non-COV-ICU-1 (n=4), non-COV-ICU-2 (n= 4), non-COV-ICU-3 (n= 3) patients were either mock- or PAO1-infected during 4hrs in serum-free DMEM free medium. Cell supernatants (diluted ½ or ¼, in duplicates) from within each category were then pooled and analyzed (49 analytes) using customized Thermofisher Luminex Human Procartaplex Mix&Match 35-plex (Cat number: PPX-35-MXXGTEK) and 14-plex (Cat number: PPX-14-MXNKTXF), according to the manufacturer’s instructions.

### Zymography

PBMCs from healthy controls (HC, n= 25), M-COV-1 (n= 19), M-COV-2 (n= 16), M-COV-3 (n= 7), COV-ICU-1 (n= 6), COV-ICU-2 (n= 6), COV-ICU-3 (n= 2), and non-COV-ICU-1 (n=4), non-COV-ICU-2 (n= 4), non-COV-ICU-3 (n= 3) patients were incubated during 4hrs in serum-free DMEM free medium. Cell supernatants (diluted ½ or ¼, in duplicates) from within each category were then pooled, incubated with Laemmli loading buffer (1.5%SDS) and loaded on pre-cast Novex 10% Zymogram Plus (Gelatin) gels for zymography analysis, a useful technique to study the effects of hydrolases *in situ*.

After electrophoresis in SDS-containing Tris buffer, the gels were washed, developed, and were stained with Coomassie blue G250. Gelatinolytic activity was then revealed following destaining (10% acetic acid, 20% methanol).

### Statistical analysis

Normality (assessed with Shapiro-Wilk or Kolmogorov-Smirnov tests) and all other statistical analysis were performed with GraphPad PRISM software 9.3.1. When normally distributed, parametric Anova was performed, followed by the appropriate multi-comparison *post-hoc* Tukey’s tests. When comparing clinical parameters from 3 groups of patients (M-COV, COV-ICU, non-COV-ICU), non-parametric Kruskal Wallis tests, followed by Dunn’s multi-comparison were performed (see **Supplementary Table S1**). Alternatively, when clinical parameters were only relevant to 2 groups of patients, t-tests followed by Mann-Whitney analysis were performed (**Supplementary Table S1**). When pool of PBMC supernatants were used, statistics were not performed and histograms represent mean +/- SD of technical replicates. For PCA and dendrogam/heatmaps analysis, an unbiased multivariate principal component analysis (PCA) global analysis, encompassing all 49 Luminex analytes (see **Supplementary Table S3**) was performed (panel A), using the ClustVis 2.0 program (https://biit.cs.ut.ee/clustvis/?s=mnEhQJZfoKkEurv). A heatmap was then obtained, using the same program (panel B).

## Results

### Healthy subjects and patient’s cohorts

Our subjects cohort was composed of healthy controls (HC, n = 25), idiopathic fibrosis patients (IPF, n=17), moderate COVID hospitalized patients (M-COV, n=58), COVID patients admitted in ICU (COV-ICU, n=48), and non-COVID patients admitted in ICU (non-COV-ICU, n=12), the latter 3 groups being assessed longitudinally (samples designated ‘-1’, ‘-2’ and ‘-3’).

IPF was chosen as a lung chronic ‘non-infectious’ disease, allowing for a comparison with COVID- and non-COVID ICU diseases, all acute and mostly infectious by nature.

As widely reported before, the hemogram of COVID patients revealed a clear increase in the NLR (neutrophil lymphocyte ratio) in the COVID patients, i.e 3.2 in ‘moderate COVID’ (M-COV) versus 9.2 in severe COVID patients (COV-ICU), compared to a normal range of 1-2 in healthy controls. All the other clinical and hematological read-outs were also well in line with the extended reported literature, with most inflammatory markers being markedly increased in the M-COV and COVID-ICU group (see **Supplementary Table S1** for a summary of these parameters).

### PBMCs FACS analysis

We first assessed by FACS (with a lymphoid and myeloid mix of antibodies, see **Supplementary Figures S1**, **S2** for the gating strategy and **Supplementary Table S2** for the antibody mix) the PBMCs of a smaller sampling of our subjects, i.e 11 HC, 6 IPF, 13 M-COV-1 patients (patients giving blood on average 10.3+/- 1.5 days after symptoms onset), 12 COV-ICU-1 patients (severe COVID patients giving blood on average 2.6 +/- 0.7 days after admission in ICU, n=12), and non-COV-ICU-1 patients (non-COVID patients giving blood on average 1.33 +/-0.3 days after ICU admission, n=4).

**Figure 1:**
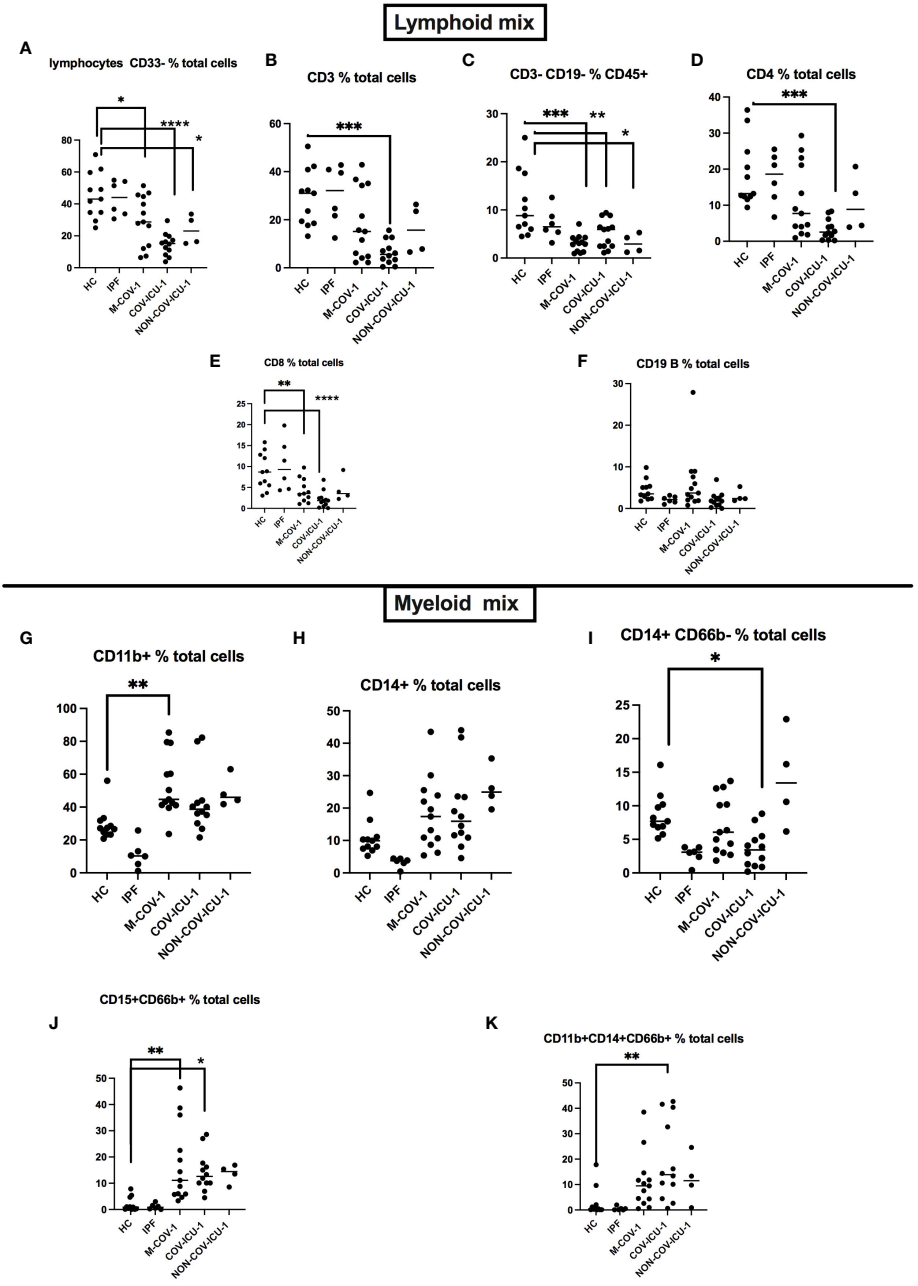
FACS analysis of PBMCs from healthy subjects and patients. PBMCs from healthy Controls (HC, n=11), idiopathic fibrosis patients (IPF, n=6), M-COV-1 patients (n=13), COV-ICU-1 patients (n=12), non-COV-ICU-1 patients (n=4) were first incubated with Fc Block antibody (BioLegend) and viability dye (Vital Amcyan au 1/1000, eBioscience) for 15 min at 4°C, washed, and stained with specific antibodies (see **Supplementary Table S2**), using both a lymphoïd mix **(A–F)** and a myeloid mix **(G–K)**. Data show mean +/- SEM. Statistical significance: Normality tests were performed for each panel and data were considered normally distributed when Shapiro-Wilk or Kolmogorov-Smirnov tests were positive for at least 3 groups out of 5 **(A–J)**. Then, multiple comparison Anova tests were performed, followed by Tukey’s test, *p<0.05, **p<0.01, ***p<0.001, ****p<0.0001. Data from **(K)** were not normally distributed and statistical significance was assessed with Kruskall Wallis, followed by Mann-Whitney tests (**p<0.01).

We showed a down-regulation of T lymphocytes (total CD3+ as well as CD4+ and CD8+, **Figures 1A, B, D, E**), and CD3-CD19- cells (likely NK cells, **Figure 1C**) in M-COV-1 and COV-ICU-1, compared to HC and IPF groups, while the B cell compartment did not exhibit any differences (**Figure 1F**). By contrast, myeloid cells taken as a whole (CD11b+; CD14+, **Figures 1G–I**), and more particularly neutrophils (CD15+CD66+, **Figures 1J, K**) were increased in the M-COV and ICU groups (both COV- and non-COV), when compared to the ‘non-acute’ groups (HC and IPF). This latter cell population will be referred in the rest of the manuscript as ‘low-density neutrophils’ (LDN), on the basis of their presence in the PBMC fraction.

### PBMCs enzymatic activities and its impact on lung epithelial cells

**Figure 2:**
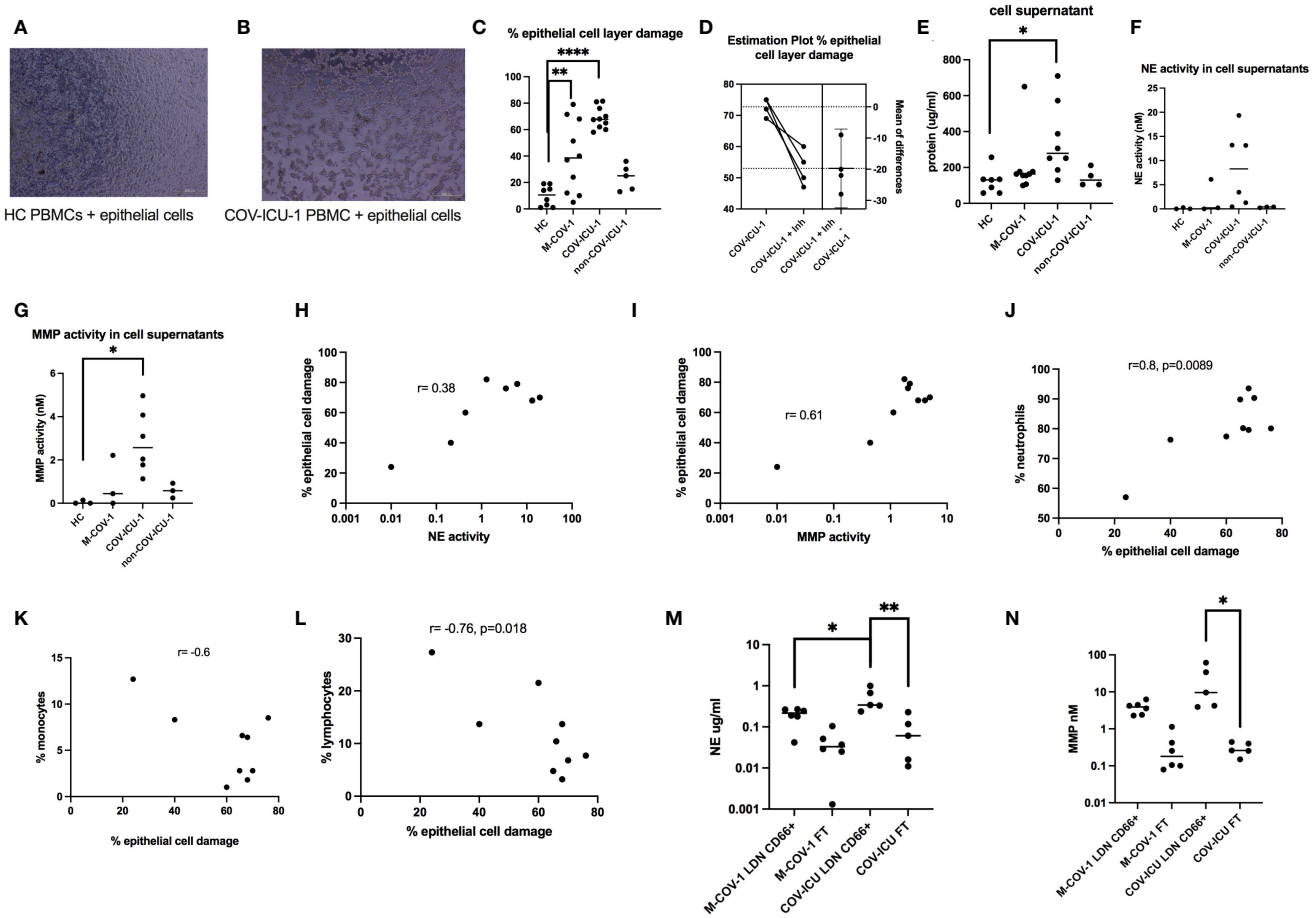
Cytotoxic effects of COVID-PMCS on lung epithelial cells. PBMCs from healthy Controls (HC, n=8), M-COV-1 patients (n=10), COV-ICU-1 patients (n=10), non-COV-ICU-1 patients (n=5) were incubated with a confluent epithelial cell layer of A549 cells during 16hrs [representative pictures are represented in **(A, B)**]. Epithelial cell damage was assessed, using the Fiji ImageJ software **(C)**, without **(C)**, or with **(D)** pre-incubation of PBMCs 4hrs with protease inhibitors (Inh = SLPI 100uM + batimastat 100uM). Supernatants were collected for LDH protein content **(E)** and enzymatic activities (NE, MMP, depicted in **F, G**). Cellular damage was shown to be positively correlated with NE and MMP activities **(H, I)**, neutrophil concentration **(J)**, and inversely correlated with the presence of monocytes and lymphocytes [(**K, L**, respectively)]. Immunomagnetic (CD66abce Miltenyi magnetic beads) selection was performed to obtain low-density neutrophils (LDN, positively selected) and a mixture of monocytes and lymphocytes (negatively selected in the column ‘flow through’, labelled ‘FT’) from M-COV (n=6) and COV-ICU PBMCs (n=5) PBMCS. NE and MMP activities were then measured in the supernatants of LDN and FT cells, 4hrs post culture **(M, N)**. Data show mean +/- SEM. Normality tests were performed for **(C–F)** and **(L, M)**, as explained in the legend of **Figure 1**. All tests showed normality except for **(E)**, where the N number was too small to conclude. Then, multiple comparison Anova tests were performed, followed by Tukey’s test, *p<0.05, **p<0.01, ****p<0.0001.

When analyzing their activity, we showed that total COVID PBMCs (both M-COV-1 and COV-ICU samples) were cytotoxic on epithelial cells, unlike ‘Control PBMCs’ and importantly unlike non-COV-ICU PBMCs (**Figures 2A–C**), as assessed by degradation of the confluent cell layer (**Figure 2C**) and release of LDH in the supernatant (**Figure 2E**). This effect was significantly inhibited by the addition of a neutrophil elastase (NE) inhibitor (100 uM SLPI) and that of a metalloprotease (MMP) inhibitor (100 uM batimastat), denoted ‘Inh’ in **Figure 2D**. In accordance, the degradation of the epithelial cell layer correlated with the concentration of these active proteases in the medium (**Figures 2F–I**), and with the original neutrophil concentration (as assessed in the patient’s hospital hemogram, **Figure 2J**), but was inversely correlated with monocytic and lymphocytic content in these same samples (**Figures 2K, L**). In addition, using CD66abce magnetic beads to separate LDNs from monocytes and lymphocytes in the PBMC fraction, we demonstrated that most of the NE and MMP activities were indeed mainly present in the LDN fraction (LDN), and not in the ‘flow through’ (FT) containing mononuclear cells (not shown) (**Figures 2M, N**).

### Sequential analysis of mediators of unstimulated PBMCs from moderate COVID (M-COV), ICU- (COV-ICU and non-COV-ICU) and IPF patients

#### Principal component and dendrogram analysis

To study further PBMC inflammatory mediators, and to potentially increase the statistical strength of our study, we decided to analyze a much larger number of PBMC samples, taken at different timings post-infection. To that effect, we constituted a pool of HC PBMCs (n=25), IPF (n=16), M-COV1 (n=19, 13 days+/-1.1 after symptoms onset), M-COV-2 (n=16, 19+/-1.4 days post-symptoms), M-COV-3 (n=7, 116+/- 4 days post symptoms, the latter being a group of patients returning to the hospital for a check-up visit), COV-ICU-1 (n=6, 4.3 +/- 1.1 days after ICU admission), COV-ICU-2 (n= 6, 11.3 +/- 2.7 days after ICU admission), COV-ICU-3 (n=2+/-, 20 +/-1 days after ICU admission), non-COV-ICU-1 (n= 4, 8.25+/- 4 days after ICU admission), non-COV-ICU-2 (n=4, 13.2+/- 4.6 days after ICU admission), non-COV-ICU-3 (n= 3, 22+/- 6 days after ICU admission).

We then analyzed longitudinally by Luminex 49 analytes [a variety of proteases, cytokines and pro/anti-inflammatory/repair mediators (see list in **Supplementary Table S3**)] from the secretomes of total steady-state PBMCs cultured 4h in serum-containing medium (see M&M).

**Figure 3:**
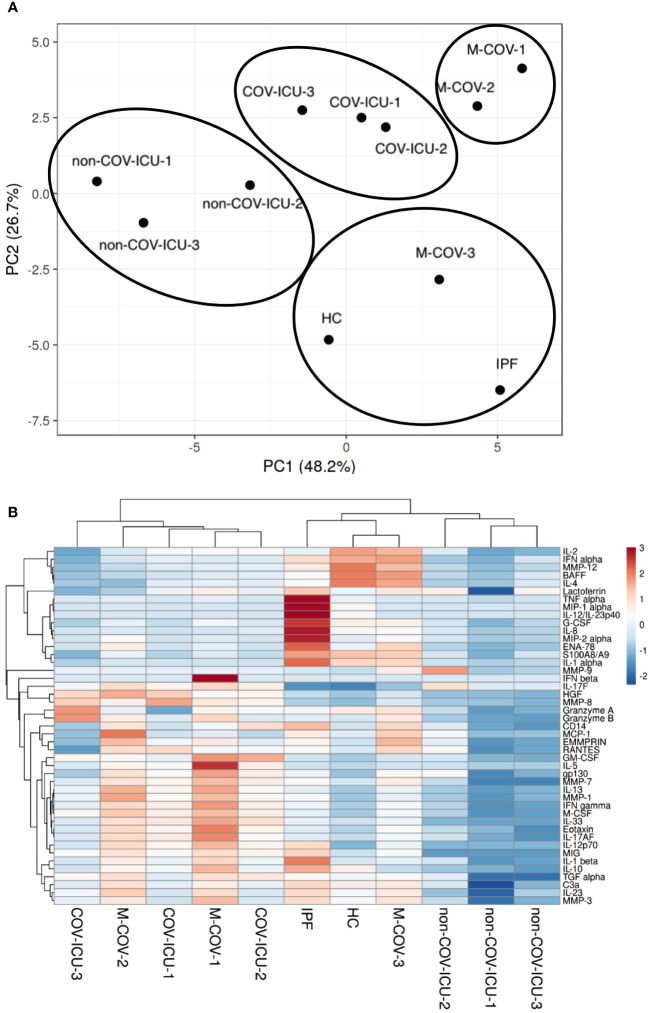
PCA and heatmap analysis of Luminex analytes from PBMCs samples (HC, IPF, M-COV, COV-ICU, non-COV-ICU). PBMCs from healthy controls (HC, n= 25), from IPF (n= 16), M-COV-1 (n= 19), M-COV-2 (n= 16), M-COV-3 (n= 7), COV-ICU-1 (n= 6), COV-ICU-2 (n= 6), COV-ICU-3 (n= 2), non-COV-ICU-1 (n=4), non-COV-ICU-2 (n= 4) and non-COV-ICU-3 (n= 3) patients were incubated 4hrs in serum-free DMEM medium. An unbiased multivariate principal component analysis (PCA) global analysis, encompassing all 49 Luminex analytes (**Supplementary Table S3**) was performed **(A)**, using the ClustVis 2.0 program (https://biit.cs.ut.ee/clustvis/?s=mnEhQJZfoKkEurv). A Heatmap was then obtained, using the same program **(B)**.

PCA and dendrogram/Heatmap analysis of these data showed (**Figure 3**) that HC (healthy controls), IPF, M-COV3 clustered together, compared to other COVIDs and non-COVID ICU patients. Notably, the COV-ICU groups discriminated from the non-COV ICU ones, demonstrating proteomic specificities in the 2 groups of ICU patients. Of note, IPF patients PBMCs clustered with HC and M-COV-3, rather than with the ‘acute’ COVID and non-COVID-ICU PBMCs, suggesting again that ‘acute ‘COVID patients had a clear signature in terms of inflammatory/immune mediators. The mediators analyzed were then, for representative purposes, arbitrarily separated into 3 categories, namely ‘proteases’, ‘myeloid/stromal mediators’ and ‘lymphocytic mediators’.

#### Unstimulated PBMCs protease output

**Figure 4:**
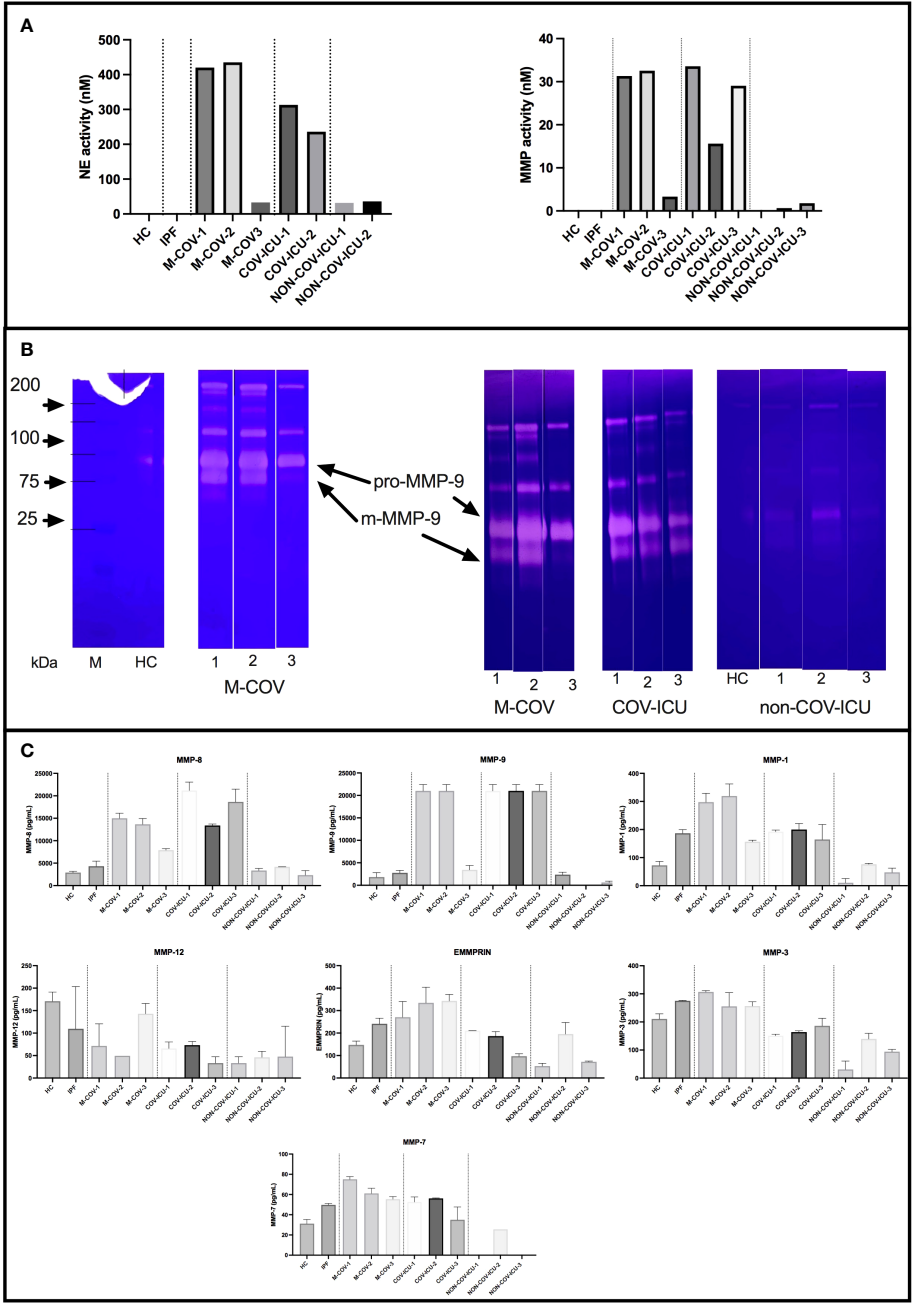
Steady state protease content and enzymatic activity of PBMCs from healthy Controls (HC), COVID (moderate and ICU) and non-COVID ICU patients. PBMCs from healthy controls (HC, n= 25), from M-COV-1 (n= 19), M-COV-2 (n= 16), M-COV-3 (n= 7), COV-ICU-1 (n= 6), COV-ICU-2 (n= 6), COV-ICU-3 (n= 2), and non-COV-ICU-1 (n=4), non-COV-ICU-2 (n= 4) and non-COV-ICU-3 (n= 3) patients were incubated during 4hrs in serum-free DMEM medium. Supernatants from each subject category were pooled together. For technical reasons, NE activity could not be assessed in NON-COV-ICU-3 samples. Cell supernatants (diluted ½ or ¼, in duplicates) from each subjects group were then pooled and analysed for NE and MMP enzymatic activities, using appropriate fluorogenic synthetic substrates **(A)**, for gelatinase activity [zymography, **(B)**], and for MMPs antigenic levels [Thermofisher Luminex Human Procartaplex Mix&Match, **C**)]. NB: M-COV samples were analysed by zymography in 2 independent experiments [**(B)**, 2^nd^ and 3^rd^ panels from the left]. Results indicate mean +/- SD from technical replicates.

When proteases were considered, we showed that all unstimulated COV PBMC samples had high pan-metalloprotease (MMP) and neutrophil elastase (NE) activities (**Figure 4A**), confirming on a much larger scale (pool of many individuals) on isolated PBMCs what was observed in the COV-PBMC-epithelial cell culture system analyzed in **Figure 2**. These activities were undetectable in HC and IPF PBMCs supernatants and extremely low in non-COV ICU samples. Within the COV group of patients, we showed that M-COV1/2 and COV-ICU MMP and NE activities were comparably high. Notably, M-COV-3 PBMC samples had very similar activities (i.e undetectable) to HC and IPF samples, suggesting a return to ‘homeostasis’ in these COVID patients.

Because of the ‘pan-MMP’ nature of the fluorogenic substrate used to assess MMP activity, we then analyzed the activity of the samples through zymography (**Figure 4B**).

Although inactive pro-MMP-9 was detectable in all the analyzed samples, the main gelatinase activity was that of mature m-MMP-9, which was present in M-COV-1/2 and COV-ICU samples, but was greatly reduced in M-COV-3, and virtually absent in HC and non-COV-ICU samples. Because gelatin zymography preferentially detects MMP-9 and MMP-2, we confirmed that MMP-9 was the main metalloprotease over-expressed in COVID samples by measuring antigenic levels of a broader range of these enzymes by Luminex (**Figure 4C**). Indeed, MMP-9 antigenic levels were by far, with MMP-8 (the latter not being a gelatinase, therefore not detected in our zymography system), the highest of all metalloproteases measured. Notably, MMP-2, the other gelatinase, was not expressed at all (not shown).

#### Unstimulated PBMCs myeloid/stromal cytokines output

**Figure 5:**
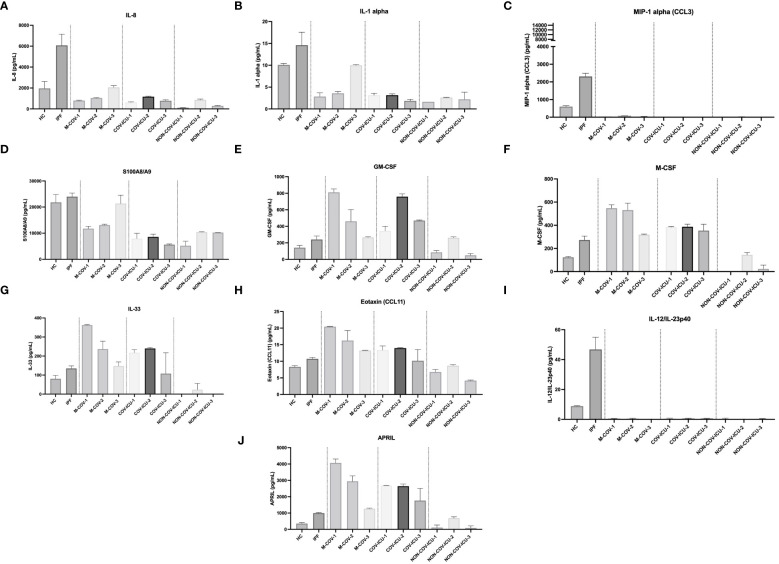
Production of steady state myeloid cytokines by PBMCs from healthy Controls, IPF, COVID (moderate and ICU) and non-COVID ICU patients. PBMCs from healthy controls (HC, n= 25), from IPF (n= 16), M-COV-1 (n= 19), M-COV-2 (n= 16), M-COV-3 (n= 7), COV-ICU-1 (n= 6), COV-ICU-2 (n= 6), COV-ICU-3 (n= 2), non-COV-ICU-1 (n=4), non-COV-ICU-2 (n= 4) and non-COV-ICU-3 (n= 3) patients were incubated 4hrs in serum-free DMEM medium. Supernatants from each subject category were pooled together. Cell supernatants (diluted ½ or ¼, in duplicates) from within each patient sub-group were then pooled and analysed (49 analytes) using customised Thermofisher Luminex Human Procartaplex Mix&Match 35-plex (Cat number: PPX-35-MXXGTEK) and 14-plex (Cat number: PPX-14-MXNKTXF). The image shows values for arbitrarily named ‘myeloid cytokines’ **(A–J)**. Results indicate mean +/- SD from technical replicates.

Among the myeloid/stromal innate immune mediators, IL-8, MIP-1α, IL-1α, S100A8/A9 levels were reduced in M-COV1/2 PBMCs (**Figures 5A–D**), compared to HC and IPF PBMCs (which overall behaved similarly), and there was always a trend for the last sequential sample (M-COV-3) behaving like HC or IPF samples (except for MIP-1a, whose levels remained low), again demonstrating a ‘return to homeostasis’ in these patients.

By contrast, and potentially related to the high myeloid content observed in these patients, the hematopoietic innate cytokines M-CSF and GM-CSF levels were higher in M-COV1/2 and COV-ICU patients (**Figures 5E, F**), compared to HC and IPF subjects and returned to almost normal values in M-COV-3 patients. Overall, the level of these cytokines stayed low in the non-COV ICU samples.

#### Unstimulated PBMCs lymphoid cytokines output

**Figure 6:**
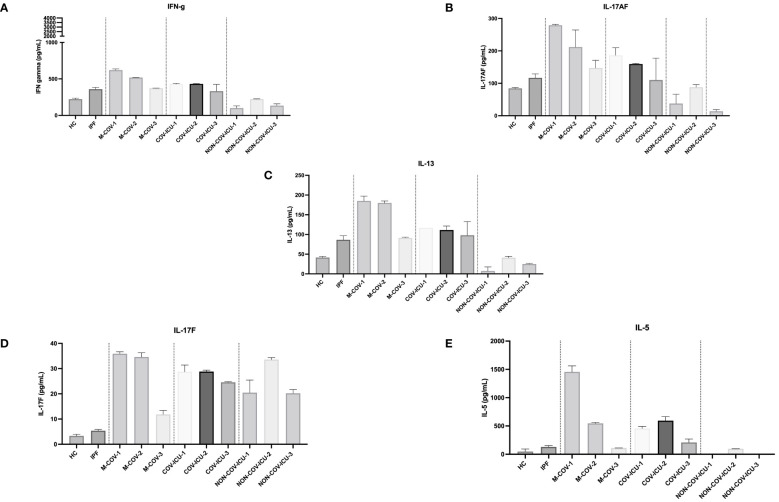
Production of steady state lymphoid cytokines by PBMCs from healthy Controls, IPF, COVID (moderate and ICU) and non-COVID ICU patients. PBMCs from healthy controls (HC, n= 25), and from IPF (n= 16), M-COV-1 (n= 19), M-COV-2 (n= 16), M-COV-3 (n= 7), COV-ICU-1 (n= 6), COV-ICU-2 (n= 6), COV-ICU-3 (n= 2), and non-COV-ICU-1 (n=4), non-COV-ICU-2 (n= 4) and non-COV-ICU-3 (n= 3) patients were incubated 4hrs in serum-free DMEM free medium. Supernatants from each subject category were pooled together. Cell supernatants (diluted ½ or ¼, in duplicates) from within each category were then pooled and analysed (49 analytes) using customised Thermofisher Luminex Human Procartaplex Mix&Match 35-plex (Cat number: PPX-35-MXXGTEK) and 14-plex (Cat number: PPX-14-MXNKTXF). The image shows values for arbitrarily named ‘lymphoid cytokines’ **(A–E)**. Results indicate mean +/- SD from technical replicates.

In contrast to myeloid/stromal cytokines, M-COV PBMCs ‘lymphoid cytokines’ were not down-regulated, when compared to the HC and IPF groups (**Figure 6**). Overall, there was a clear ‘type 2/type 3 signature’ present in these samples, with high levels of IL-13, IL-17F, IL-17AF, IL-5 in M-COV1/2 samples (**Figure 6**), echoing the equally high levels of ‘type 2’ APRIL, IL-33, eotaxin found in the ‘myeloid/stromal’ group (**Figures 5G, H, J**). Again, these lymphoid cytokines were drastically decreased in M-COV-3 samples (**Figure 6**). Notably, by contrast, the levels of IL-5, IL-17AF, IL-17F, remained high in COV-ICU samples and the latter was also notably increased in non-COV-ICU samples (**Figure 6**).

Overall, our results showing a down-regulation of key myeloid markers on COVID-PBMCs, associated with an ‘unfavorable’ Th2 biasing of lymphoid cytokines suggested that this phenotype may be conducive to a greater sensitivity/lesser responsiveness to bacterial microbes such as *Pseudomonas aeruginosa* (*P.a*), an important nosocomial pathogen in ICU settings, and in COVID patients, as demonstrated previously (33–36), and in the present study (**Supplementary Tables S4**-**S6**).

#### Live PAO1-infected PBMCs myeloid/stromal cytokines output

**Figure 7:**
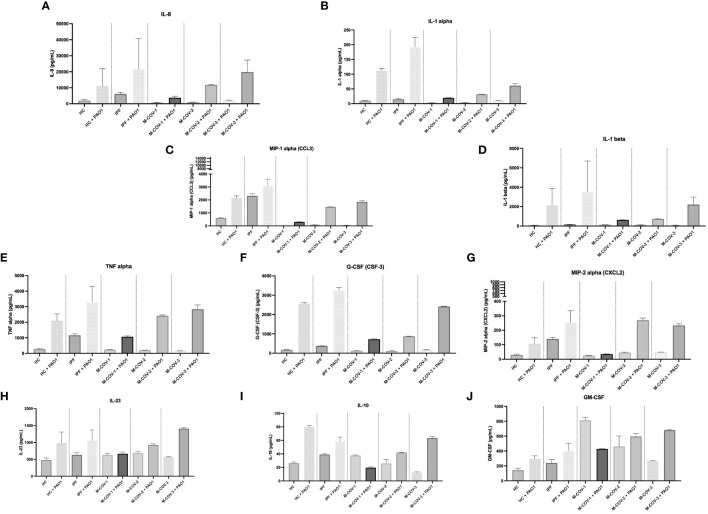
Production of myeloid cytokines by PBMCs (+/- PAO1) from healthy Controls, IPF, and M-COVID (moderate). PBMCs from healthy controls (HC, n= 25), and from IPF (n= 16), M-COV-1 (n= 19), M-COV-2 (n= 16), M-COV-3 (n= 7) patients were either mock (medium) or co-cultured with PAO1 (moi 1) during 4hrs in serum-free DMEM free medium. Supernatants from each subject category were pooled together. Cell supernatants (diluted ½ or ¼, in duplicates) from within each category were then pooled and analysed (49 analytes) using customised Thermofisher Luminex Human Procartaplex Mix&Match 35-plex (Cat number: PPX-35-MXXGTEK) and 14-plex (Cat number: PPX-14-MXNKTXF). The image shows values for arbitrarily designed ‘myeloid cytokines’ **(A–J)**. Results indicate mean +/- SD from technical replicates.

Indeed, compared to HC and IPF patients (which again overall behaved similarly), M-COV1/2 PBMCs secreted, upon infection with live PAO1, less IL-8, MIP-1α, IL-1α, IL-1b, TNF-α, G-CSF, MIP-2α, IL-23 and IL-10 (**Figures 7A–I**). Again notably, and consistently, M-COV-3 PBMC behavior was clearly different in that they overall recovered their response to the bacterium, showing a return to ‘baseline values’. Interestingly, GM-CSF (**Figures 7J**) basal levels were reduced by PAO1 infection in M-COV-1 PBMCs, but not in M-COV-2 and M-COV-3 samples.

Among the COV-ICU myeloid/stromal mediators, there was, post-PAO1, a reduced induction of C3a, ENA-78, G-CSF, IL-1β, IL-8, MIP-1α, IL-1α (**Supplementary Figure S4**). This was at variance with the PBMCs from the convalescent M-COV-3 group, where cytokine levels had returned to normal (**Figure 7**), clearly demonstrating a sustained de-activation of potential anti-bacterial responses in the individuals kept in ICU. Conversely, and notably, HGF levels remained high in COV-ICU PBMCs. Similarly to the M-COV groups discussed above, M-CSF and GM-CSF levels remained high in COV-ICU patients (but not in non-COV-ICU ones), compared to C and IPF subjects, again in keeping with the hematopoietic function of these cytokines (**Supplementary Figure S4**).

In the non-COV-ICU groups, the most salient result was that MIP-1α levels were increased compared to COV-ICU corresponding groups (**Supplementary Figure S4**). Also importantly, similarly with the COV-ICU group, PBMCs secreted less ENA-78, G-CSF, IL-1β, IL-8, MIP-1α, IL-1α in response to PAO1, demonstrating that the ‘deactivation’ of innate responses is not specific to COVID patients, but is also present, more generally, in ICU patients (**Supplementary Figure S4**).

To assess which cell type within the PBMC fraction was responsive (or less) to PAO1, LDN and FT cells (obtained from CD66abce Miltenyi magnetic beads columns, see above) were separately infected, and IL-1b, TNF-α, and IL-8 protein output was measured. Neither HC nor M-COV-1 LDNs responded to PAO1, whereas FT cells (almost certainly monocytes) from HC PBMCs (but not from M-COV-1 or ICU-COV cells) secreted high levels of these cytokines (**Supplementary Figure S3**).

#### Live PAO1-infected PBMCs lymphoid cytokines output

In contrast to myeloid cytokines, PAO1 induced no or little amounts of ‘lymphoid cytokines’ from HC and IPF PBMCs and instead down-regulated (except for IL-17F and IL-4) the level of basally-secreted cytokines in M-COV-1/2 PBMCs (notably Granzyme A, IL-13, IL-17A/F, IL-5 (not shown).

Similarly to M-COV PBMCs, lymphoid cytokines overall were not down-regulated post-PAO1 in the ICU group (both COV- and non-COV), particularly IFN-g, IL-17AF, IL-5, GranzA, GranzB, whose levels remained high in PBMCs from patients staying in ICU for a longer period of time (COV-ICU-3, **Supplementary Figure S5**).

#### T cell proliferation

**Figure 8:**
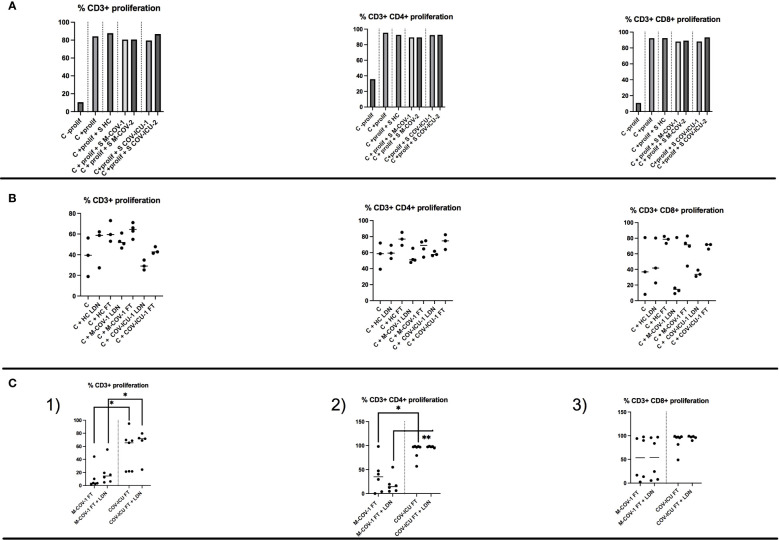
T cell proliferation in total PBMCs and in the FT-PBMC fraction of HC subjects and COVID patients. **(A)** The ‘pool of supernatants’ (S) from PBMCs of each group of subjects (HC, M-COV1/2, COV-ICU1/2) analysed previously (**Figures 5**–**7**) were incubated 2hrs at 37°C with CTV-labelled PBMCs from an unrelated HC donor (Control=C). After stimulation with 20ng/ml rIL-2 plus anti-CD3 and anti-CD28 Dynabeads, cell proliferation (C + prolif +/- S) was assessed by FACS analysis as detailed in the Supplemental M&M section, and compared to HC C + prolif alone. **(B)** Either low-density neutrophils (LDNs) or FT cells (monocytes and lymphocytes), purified from respectively HC subjects (n=3), M-COV-1 (n=3) and COV-ICU-1 (n=3) patients were incubated with CTV-labelled PBMCs from an unrelated HC donor (Control=C). Stimulation of cells and allogenic proliferation (during 4 days) analysis were performed as above. **(C)** CTV-labelled FT cells (monocytes and lymphocytes) were purified from M-COV-1 and COV-ICU PBMCs, and stimulated for proliferation during 4 days (same protocol as above), alone or with syngenically purified low-density neutrophils/LDNs from the same COVID individuals. Stimulation of cells and syngenic proliferation (during 4 days) analysis were performed as above. Statistical significance: normality tests were performed for panels **(B, C)**, as explained in the legend of **Figure 1**. Normality was achieved for panels **(B)**, and multiple comparison Anova tests showed no statistical significance. Data in panels **(C)** were showed not to be normally-distributed, and statistical significance was assessed with Kruskall Wallis, followed by Mann-Whitney tests (*p<0.05, **p<0.01).

Although T cell numbers are clearly reduced in COV-PBMCs (**Figure 1** and the extended literature), T cell ‘signature’ cytokines did not globally follow the same trend (see above), suggesting that their phenotype is clearly different. We first showed that the ‘pool of supernatants’ (S) from each group of subjects (HC, M-COV1/2, COV-ICU1/2) analyzed previously (**Figures 5**–**7**) had no inhibitory effect on exogenous HC PBMCs (C prolif) proliferation, when activated with an anti-CD3/anti-CD28/IL-2 mix, used as a ‘pan/non specific’ stimulus of lymphocyte activation (**Figure 8A**). We then found that there was a trend (although not statistically significant) for increased proliferation of labelled HC PBMCs, when stimulated with unlabeled allogenic lymphocytes (present in the FT fraction obtained from CD66abce columns, see above), but this trend was independent of the stimulating lymphocytes phenotype (HC, M-COV, ICU-COV, **Figure 8B**). Finally, to specifically assess the phenotype of COVID LDNs, we demonstrated that the latter (obtained as above) did not reduce the proliferation of T cells present in the syngeneic FT fraction (obtained as above) from the same individuals (**Figure 8C**). Interestingly, the proliferation of ‘FT lymphocytes’ or ‘FT lymphocytes + LDNs’ was consistently higher in COV-ICU-1 than in M-COV-1 patients. Of note, for all these studies, and for technical reasons, we concentrated on the role of LDNs and could not therefore compare the phenotypic characteristics of ‘normal density neutrophils’ (NDN) in COVIDs and HC individuals.

## Discussion

Low-density neutrophils (LDN) are cells found in the PBMC fraction of blood samples after isolation from density gradient centrifugation (8–11). They have been associated with a variety of conditions including cancer and auto-immunity, but relatively little is known of their role during infections. Although they are commonly thought to be associated with an anti-inflammatory/immunosuppressive phenotype, when compared to normal density neutrophils [NDNs, (39–43)], they have also been described as having pro-inflammatory properties in diseases such as systemic lupus erythematosus (SLE), with increased type I interferon, NET production and Th1-biasing properties (44, 45). More recently, they also have been described in patients with cystic fibrosis (11, 46). In fact, they may not be as specific as original thought and they have been shown to have some overlapping functions with normal density neutrophils [NDN, (11)].

In the context of COVID-19, numbers of studies (including the present one) showed high numbers of neutrophils in COVID-19 patients, associated with lymphopenia (1–7). However, most studies have dealt with the phenotypic characterization of these cells, using principally antibody markers (FACS analysis) and relatively few have assessed the function of PBMCs and neutrophils in COVID.

Here, we show that PBMCs from both hospitalized/moderate (M-) and ICU-COVID patients, as well as non-COV-ICU patients have an increased proportion of low-density neutrophils (LDNs), associated with a decreased proportion of lymphocytes (**Figure 1**), when compared to PBMCs from healthy controls and IPF patients. Although IPF is clearly not an acute pathology, it was nevertheless deemed interesting here to include an unrelated ‘chronic lung disease’ group of patients, in addition to the more comparable non-COV-ICU group.

Irrespective, the cellular changes observed in COVID patients were associated with a high spontaneous release of active proteases such as NE and MMP (of which MMP-9 was prevalent), which we confirmed were produced by the LDN portion of PBMCs (**Figures 2L, M**), and which were positively associated with epithelial lung cell damage in *ex-vivo* experiments (**Figures 2A–H**). Relatedly, Yan Q et al. showed that early COVID-19 PBMCs had increased transcriptional levels of MMP-9 and NE, which decreased to near-normal levels in covalescent patients (47).

Importantly, although neutrophils were similarly abundant in M-COV, COV-ICU and non-COV-ICU PBMCs, they were not similarly activated. Indeed, only COVID PBMCs demonstrated a high spontaneous release of active proteases (NE and MMP), which was partly responsible of lung epithelial cell damage (**Figures 2C–F**). This clearly demonstrates that COVID PBMC LDN should not be considered as only immunosuppressive but also as potentially deleterious since highly ‘spontaneously’ activated. Mechanistically, it is generally accepted that no single stimulus is able on its own to activate neutrophils as to induce degranulation and release of proteases (48–50), and that a two-step prime/activation system is necessary, involving microbial (LPS, FMLP…) and host mediators (GM-CSF, IL-8, IL-17…). Here, GM-CSF and M-CSF stood out as suitable candidates since they were significantly increased in COV-PBMCs supernatants, compared to the other groups, including non-COV-ICU (**Figure 5**). This potential lack of priming in the latter group might explain why, despite high levels of neutrophils (**Figure 1**), lower levels of proteases and epithelial damage was observed (**Figure 2**). Caution is however warranted when comparing neutrophil-derived proteases in patients at different stages of the infectious/repair process. Indeed, whereas blood was on average sampled at days 12/20 post symptoms in M-COV-1/2 patients, it was obtained 4/11/20 days after ICU admission (i.e way beyond the symptoms appearance) in severe COV patients and 8/13/22 days in severe non-COV ICU. Indeed, proteases, and in particular MMPs have been shown to have dual activity, some promoting matrix degradation, whereas others are implicated in tissue repair/fibrosis (see ref (51) for a review). In that context, in IPF PBMC supernatants, the overall NE and MMP enzymatic activities was similarly undetectable as those of HC samples, whereas they were very high in M-COV and COV-ICU samples (**Figure 4A**), and assessing whether protease activities are only deleterious (as shown in co-culture experiments with epithelial cells, **Figure 2**), or may have a beneficial role in promoting repair is a difficult exercise. It is important to note, however, that proteases may not be the only mediators of epithelial cell damage. For technical reasons linked to the paucity of material available, we could not test the involvement of ROS or NETs, which have been shown in other studies to also be cytotoxic against epithelial cells (52–54).

Regardless of the mechanism, although these data strongly suggested that COVID LDNs are hyper-activated and potentially harmful to the alveolar septum *in vivo*, whether this has a bearing towards microbial responses has seldomly been addressed mechanistically. Indeed, even though clinical data exist to show that SARS-CoV-2 infection is associated with increased bacterial infections (33–36), mechanistic studies to assess whether total PBMCs or neutrophils/LDNs are more/less responsive towards further bacterial stimuli are sparce, and results are contradictory. For example, McLeish et al. (7) showed that phagocytosis of killed, labelled, and opsonized *S.aureus* is increased in COVID neutrophils. Similarly, Masso-Silva et al. (55) demonstrated increased phagocytosis of *S.aureus* bioparticles. By contrast, Schulte-Shrepping et al. (20) showed that neutrophils from severe COVID-19 had reduced respiratory activity (with no change in phagocytosis) after stimulation with killed, labelled and opsonized *E.coli*, and Peyneau et al. reported a decrease in zymosan particles uptake in COVID neutrophils and monocytes, indirectly suggesting that phagocytosis might be impaired in COVID (56). Also, few studies have shown that COVID blood-derived monocytes and DCs had reduced inflammatory cytokines output, following bacterial ligands (LPS, flagellin.) exposure (21, 23), but data reporting experiments with live bacteria are to our knowledge sparce.

We showed here that indeed PBMCs from COVID patients (as well as non-COV ICU patients) exhibit an impaired response to *ex-vivo* live *Pseudomonas aeruginosa* infection (a bacterium shown to be particularly present in the microbiome of SARS-CoV-2-infected patients and often present also in ICU patients, 33-36). In particular, we demonstrated a decrease in the production of protective myeloid cytokines involved in bacterial defenses (**Figures 5**, **7**, **Supplementary Figures S3**, **S4**). By contrast, lymphoid cytokines (in particular type 2/type 3) levels remained high (**Figures 6**, **Supplementary Figure S5**), both basally and post PAO1 infection, reflecting their unimpaired capacity to proliferate, when stimulated with non-specific agonists (**Figure 8**). Of note, shortage of samples prevented us from assessing direct bacterial killing (as studied in other studies mentioned above), or lymphocytic proliferation in responses to specific agonists such as *P.a* antigens. Notwithstanding this, and in agreement with Hardisty et al. (11), our data clearly demonstrate that LDNs should not be considered ‘in principle’ as intrinsically ‘inhibitory’ or ‘regulatory’, since we showed (**Figure 8**) that neither COVIDs LDNs nor monocytes were able to inhibit lymphocyte proliferation in syngeneic or allogenic systems [instead, COV-ICU lymphocytes showed even increased proliferation when compared to M-COV lymphocytes (**Figure 8C**)]. Indeed, we showed that they can also be considered as being ‘pro-inflammatory’ (at least in COVID), given their destructive potential on lung epithelial cells (**Figure 2**). In that context, although the medication received by the patients should be taken into account, we believe it unlikely that the use of corticosteroids (given to 62% and 77% of M-COV and COV-ICU patients, respectively, see **Supplementary Table S1**) would explain our results. Indeed, these drugs are known to down-regulate lymphocyte proliferation and neutrophil degranulation (57), 2 read-outs not affected in our study. In addition, a higher % of COV-ICU patients received dexamethasone, compared to M-COV patients, and yet, this was not reflected in reduced degranulation, protease content, and epithelial damage, where the opposite was found (**Figures 2**, **4**), nor in pro-inflammatory cytokines production (eg **Figure 5**) or lymphocytic proliferation (**Figure 8C**, where again the opposite was true).

In conclusion, it is also worthwhile stressing out that, as explained in the M&M section, the Luminex-based experiments have been performed pooling samples from a number of patients within ‘each category’ (HC, M-COV-1/2/3, COV-ICU1/2/3, non-COV-ICU1/2/3). Although analyzing such a wide array of cytokines would not have been possible using individual samples, this is a limitation of our study, given the known heterogeneous responses from such patients.

Notwithstanding this, we demonstrate here that COVID PBMCs are doubly harmful, through LDN-mediated lung epithelial degradation and monocytic-driven impaired responsiveness against bacterial infection, such as *P.aeruginosa*. Whether this is caused by lack of TLR- (or other receptors) mediated signaling might be an interesting avenue to explore and may explain the high prevalence of bacterial super-infections and ensuing ARDS found in COVID patients hospitalized in ICUs.

## Data availability statement

The raw data supporting the conclusions of this article will be made available by the authors, without undue reservation.

## Ethics statement

The studies involving humans were approved by “CPP Ile-de-France VI”, #2020-A00256-33. The studies were conducted in accordance with the local legislation and institutional requirements. The participants provided their written informed consent to participate in this study.

## Author contributions

CG: Writing – review & editing, Methodology, Investigation. MB-B: Writing – review & editing, Formal analysis, Methodology, Investigation. BV: Writing – review & editing, Methodology, Investigation. MJ: Writing – review & editing, Resources. DF: Writing – review & editing, Resources. J-FT: Writing – review & editing, Resources. AT-D: Writing – review & editing. PM: Writing – review & editing. BC: Writing – review & editing. IG-V: Writing – review & editing. J-MS: Writing – review & editing, Writing – original draft, Validation, Supervision, Software, Resources, Project administration, Methodology, Investigation, Funding acquisition, Formal analysis, Data curation, Conceptualization.
