# Supplementary material for: COVID-19 PBMCs are doubly harmful, through LDN-mediated lung epithelial damage and monocytic impaired responsiveness to live Pseudomonas aeruginosa exposure
Source: Front Immunol. 2024 May 21;15:1398369. doi: 10.3389/fimmu.2024.1398369 (PMC11148249; doi:10.3389/fimmu.2024.1398369)
Supplement: Supplementary file 1 [file DataSheet_1.docx]

**Supplemental Fig legends**

**Table S1 :** Demographic and clinical data of COVID patients

When comparing clinical parameters from 3 groups of patients (M-COV, COV-ICU, non-COV-ICU), Kruskal Wallis tests, followed by Dunn’s multi-comparison were performed (see Table S1). Alternatively, when clinical parameters were only relevant to 2 groups of patients, t-tests followed by Mann-Whitney analysis were performed. *p<0.05, **p<0.01, ***p<0.001, ****p<0.0001.

**Table S2 :** FACS antibodies used in the analysis of PBMCs from the subjects studied.

**Table S3 :** Mediators analysed by Luminex Human Procartaplex Mix&Match 35-plex (Cat number: PPX-35-MXXGTEK) and 14-plex (Cat number: PPX-14-MXNKTXF).

**Table S4 :** Bacterial presence in individual COVID-ICU patients samples (%)

**Table S5 :** Bacterial presence in overall COVID-ICU patients samples (%)

**Table S6 :** Bacterial presence in at least one respiratory tract sample (nose, trachea, lung) in

COVID-ICU patients samples (%)

**Table S7 :** Demographic and clinical data of IPF patients

**Figs S1-S2** : Gating strategy for the detection of monocytes, neutrophils and lymphocytes in PBMCs from healthy controls and patients (IPF, M-COV, COV-ICU, non-COV-ICU)

Singlets from a FSC/SSC lymphocytic gate were first selected, then gated for viability, followed by myeloid and lymphoid cell characterization using myeloid

(CD14 FITC 1/10 ; CD11b APC/Cy7 1/100 ; CD15 BV510 1/100 ; CD16 BV711 1/200 ; CD66b PE-Cy7 1/100), and lymphoid mixes (CD3 FITC 1/50 ; CD4 APC/Cy7 1/100 ; CD8 BV650 1/100 ; CD33 BV711 1/100 ; CD19 PE-Cy7 1/100), respectively. See Material and Methods for the detailed procedure.

**Fig S3** : **Production and secretion of IL-1b, TNFa, and IL-8 by LDN and monocytes (FT fraction) from HC and ICU-COV M-COV-1, post- infection with PAO1**

PBMCs from HC subjects (n=4), M-COV-1 patients (n=4) and COV-ICU patients (n=4) were used. From these PBMCs, 200,000 cells purified low-density (LDN) neutrophils, and a mix of 200,000 monocytes and lymphocytes (from the column flow through (FT), see legend of Fig 2) were infected with PAO1 (moi 1) during 4hrs in 96 well plates. Cell supernatants were then harvested for cytokine measurements by ELISA. Statistical significance :

Normality tests were performed for each panel and data were tested for normal distribution with Shapiro-Wilk or Kolmogorov-Smirnov tests.

Data for IL-1b were found to ne normally distributed and multiple comparison Anova tests were performed, followed by Tukey’s test, *p<0.****p<0.0001. Data for TNF a and IL-8 were found to be non-normally distributed and statistical significance was assessed with Kruskall Wallis, followed by Mann-Whitney tests, *p<0.05, ****p<0.0001.

**Fig S4 :** Production of myeloid cytokines by PBMCs from healthy Controls, COV-ICU (1-3) and non-COV-ICU (1-3) patients, mock-treated or infected with PAO1

PBMCs from healthy controls (HC, n= 25), IPF (n= 16), COV-ICU-1 (n= 6), COV-ICU-2 (n= 6), COV-ICU-3 (n= 2), non-COV-ICU-1 (n=4), non-COV-ICU-2 (n= 4) and non-COV-ICU-3 (n= 3) patients were mock-treated or PAO1-infected (moi 1) during 4hrs in serum-free DMEM medium. Cell supernatants (diluted ½ or ¼, in duplicates) from each subjects group were then pooled and analysed (49 analytes) using customised Thermofisher Luminex Human Procartaplex Mix&Match 35-plex (Cat number: PPX-35-MXXGTEK) and 14-plex (Cat number: PPX-14-MXNKTXF). Fig S4 shows values for arbitrarily named ‘myeloid cytokines’ and error bars represent SD from technical replicates.

**Fig S5 :** Production of lymphoid cytokines by PBMCs from healthy Controls, COV-ICU (1-3) and non-COV-ICU (1-3) patients, post-infection with PAO1

PBMCs from healthy controls (HC, n= 25), IPF (n= 16), COV-ICU-1 (n= 6), COV-ICU-2 (n= 6), COV-ICU-3 (n= 2), non-COV-ICU-1 (n=4), non-COV-ICU-2 (n= 4) and non-COV-ICU-3 (n= 3) patients were mock-treated or PAO1-infected (moi =1) during 4hrs in serum-free DMEM medium (moi =1). Cell supernatants (diluted ½ or ¼, in duplicates) from each subjects group were then pooled and analysed (49 analytes) using customised Thermofisher Luminex Human Procartaplex Mix&Match 35-plex (Cat number: PPX-35-MXXGTEK) and 14-plex (Cat number: PPX-14-MXNKTXF). Fig S5 shows values for arbitrarily named ‘lymphoid cytokines’ and error bars represent SD from technical replicates.
