# Supplementary figures and images for: COVID-19 PBMCs are doubly harmful, through LDN-mediated lung epithelial damage and monocytic impaired responsiveness to live Pseudomonas aeruginosa exposure
Source: Front Immunol. 2024 May 21;15:1398369. doi: 10.3389/fimmu.2024.1398369 (PMC11148249; doi:10.3389/fimmu.2024.1398369)

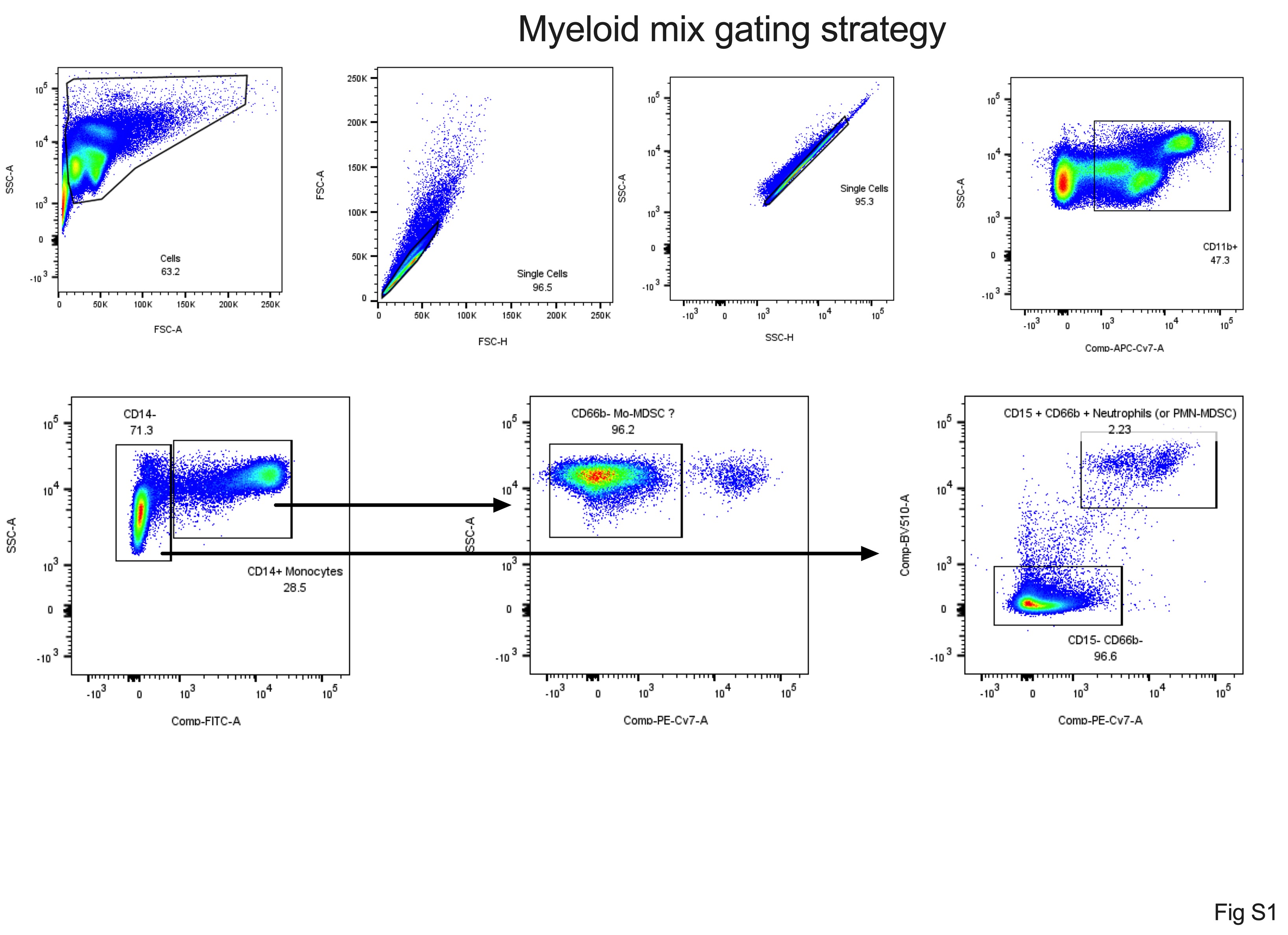

Supplement: Supplementary file 2 [file Image_1.jpeg]

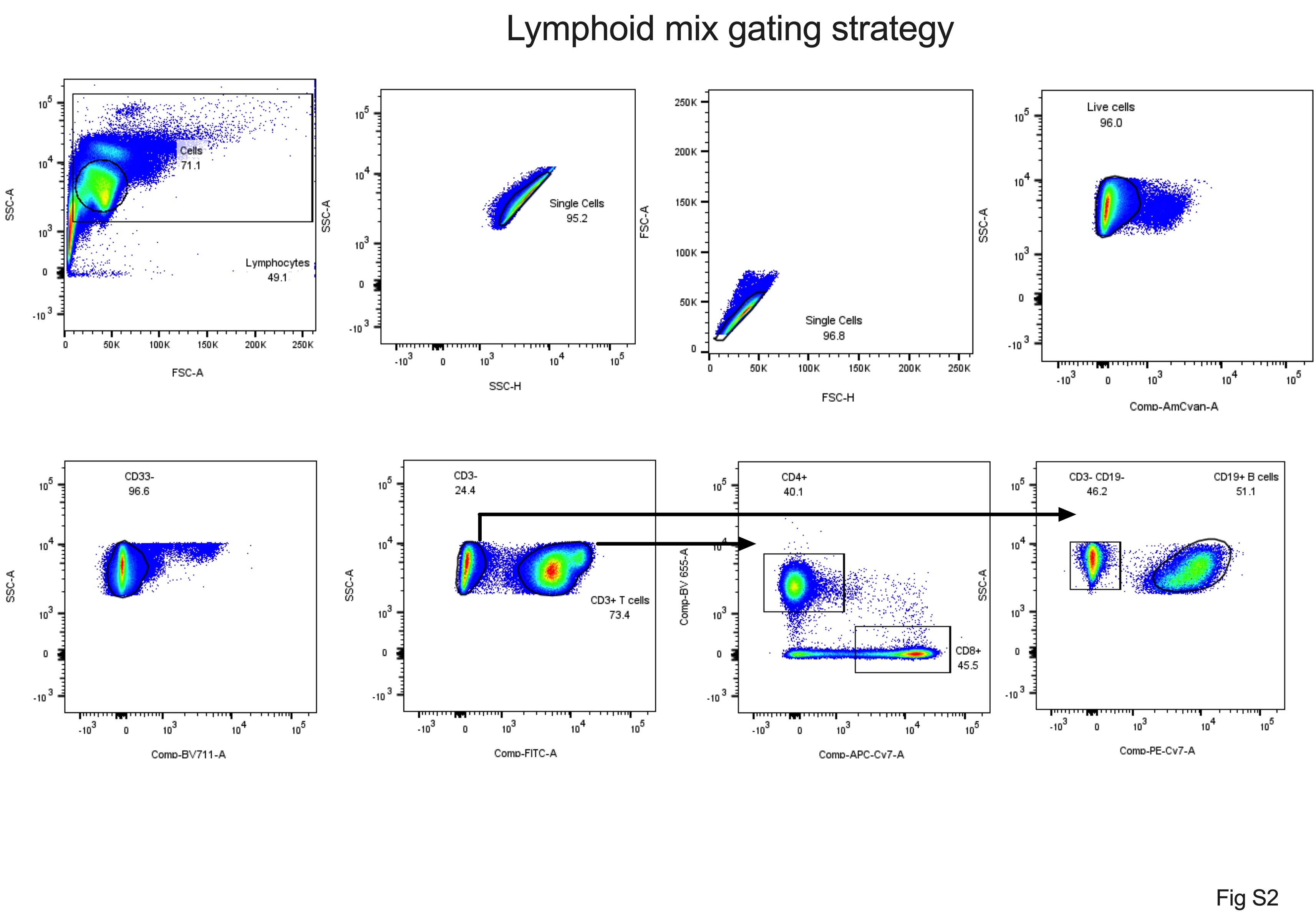

Supplement: Supplementary file 3 [file Image_2.jpeg]

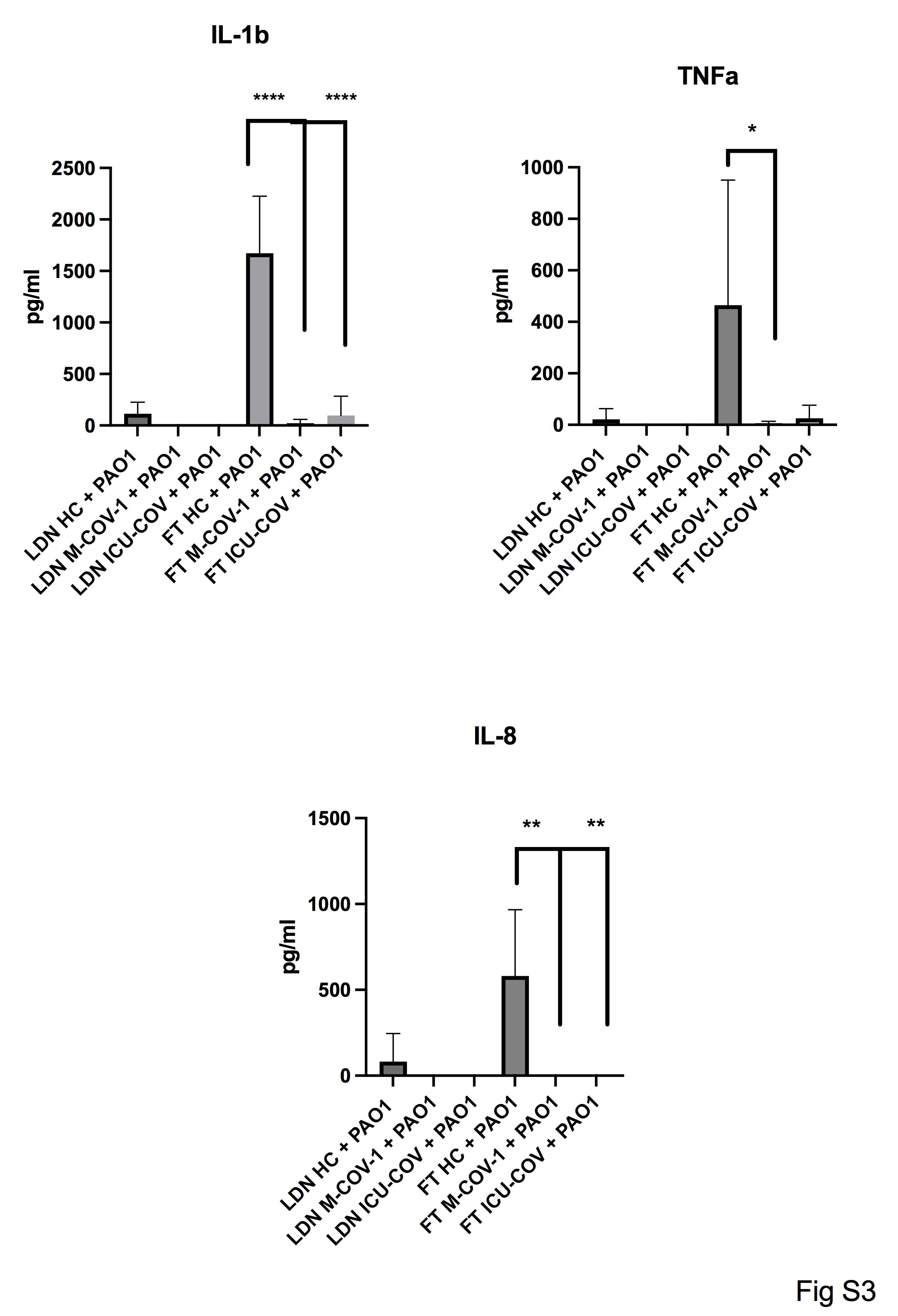

Supplement: Supplementary file 4 [file Image_3.jpeg]

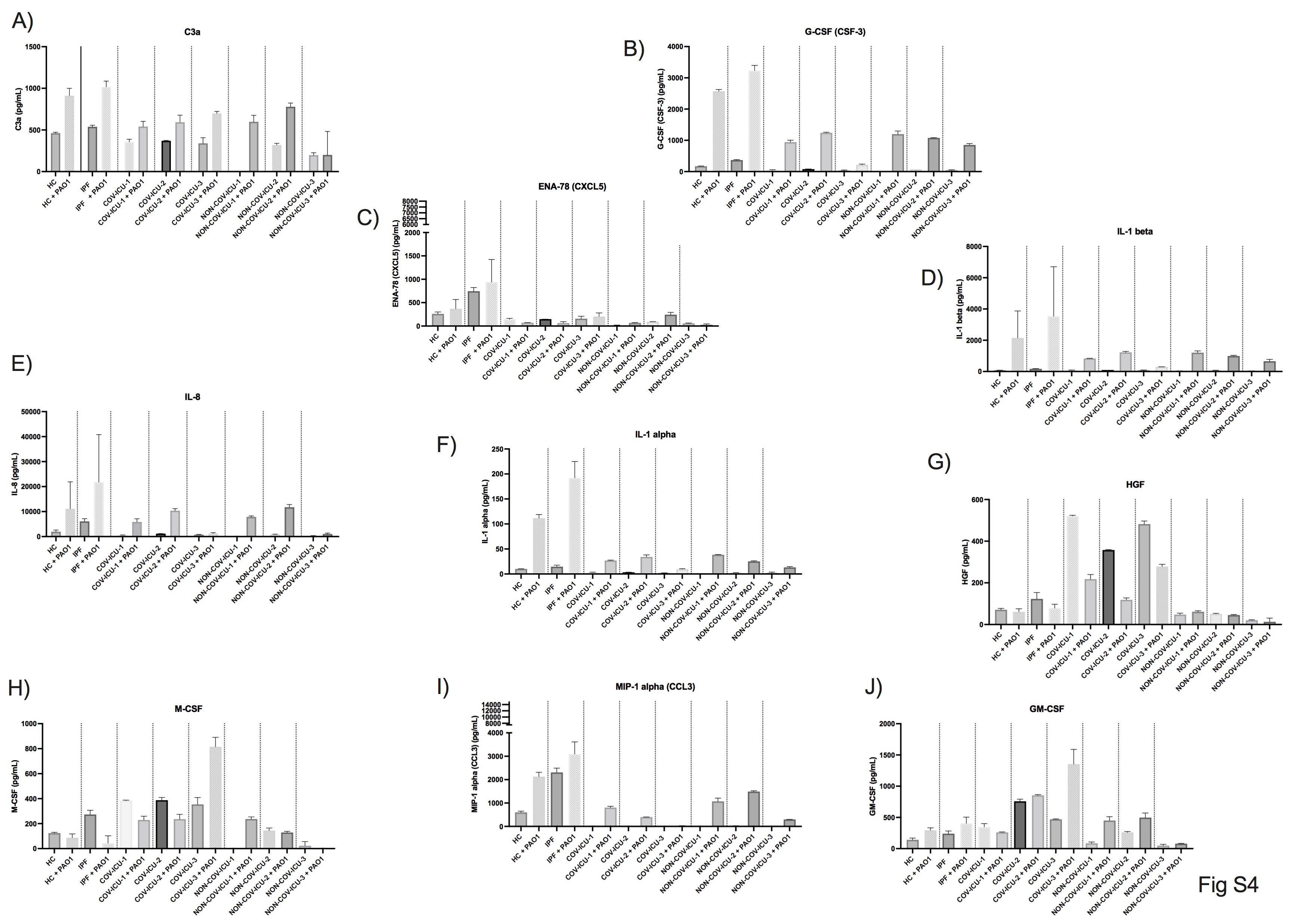

Supplement: Supplementary file 5 [file Image_4.jpeg]

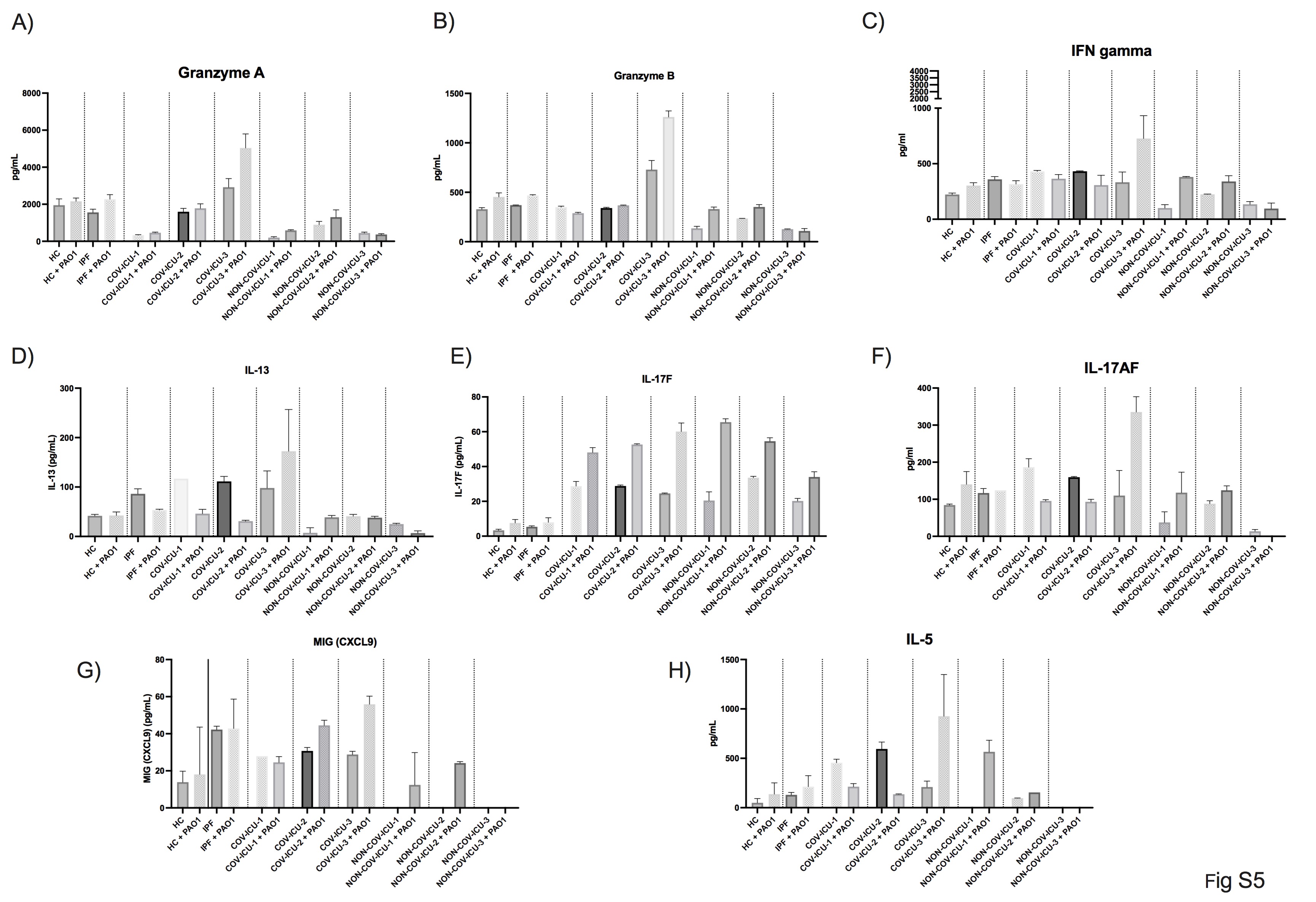

Supplement: Supplementary file 6 [file Image_5.jpeg]
