# Supplementary material for: COVID-19 PBMCs are doubly harmful, through LDN-mediated lung epithelial damage and monocytic impaired responsiveness to live Pseudomonas aeruginosa exposure
Source: Front Immunol. 2024 May 21;15:1398369. doi: 10.3389/fimmu.2024.1398369 (PMC11148249; doi:10.3389/fimmu.2024.1398369)
Supplement: Supplementary file 7 [file Table_1.docx]

|  | **M-COV (hospitalized)** | **COV-ICU** | **non-COV-ICU** |
| --- | --- | --- | --- |
| **Patients, n** | 58 | 48 | 12 |
| **Age, years** | 60 (24-86) | 62.5 (36-80) | 60 (38-77) |
| **BMI, kg/m^2^** | 28 (18-49) | 30 (23-51) | 25 (16-34) |
| **Sex, male (%)** | 74% | 69% | 67% |
| **PaO_2_/FiO_2_ ratio at blood sample, mmHg** | NA | 136.5 (51-404) | 275.4 (67-506) ¶ |
| **Oxygen flow at blood sample, l/min** | 5 (0-15) | NA | NA |
| **Comorbidities**  *Diabetes*  *Hypertension*  *Chronic lung disease* | 33%  47%  31% | 31%  48%  12.5% | 8%  17%  33% |
| **Laboratory values at admission** |  |  |  |
| ***Blood cells***  *Total leukocytes (G/l)*  *lymphocytes (G/l)*  *lymphocytes (% total leucocytes)*  *neutrophils (G/l)*    *neutrophils (% total leucocytes)*  *monocytes (G/l)*    *monocytes (% total leucocytes)* | 6.68 (2.2-13)    1.1 (0.2-12.7)  16.4  5.1 (1.3-8.3)  76  0.5 (0.06-1.6)  7.4 | 8.95 (1.8-30.7)*    0.6 (0.2-2.89) *  6.7**  7.61 (1.3-15.18) *  85*  0.36 (0.08-4.51)  4** | 20.33 (9.7-33.12)**** ¶¶  1.28 (0.38-9.35)    6.3*  18.75 (6.98-30.07) **** ¶  92.2** ¶¶¶¶  0.71 (0.29-1.52) ¶  3.4 |
| ***Blood markers***  *C-reactive protein, mg/l*  *ALAT, UI/l*  *ASAT, UI/l*  *LDH, UI/l*  *Creatinine, micromoles/l*  *Ferritin, µg/l*  *NT-proBNP, ng/ml*  *D-Dimer, ng/ml* | 106 (3-395)  36 (13-162)  38.5 (10-161)  354.5 (5-568)  72 (39-127) 601(252-4412)  140 (6-3413)  939 (377-5526) | 158 (52-564)  40.5 (17-530)  43 (14-478)  377.5 (306-745)  67 (25-382)  1884 (191-3603)  373 (13-4173)  1184 (296-27720) | 66.5 (2-337)  30.5 (19-1152)  58 (16-1506)  304 (154-1139)  94.5 (54-451)  242 (242-2577)  1616 (154-7885) **  3882.5 (444-35000) |
| **Virus load (Ct)** | 28.5 (16.6-38) | 24.3 (19-34) | NA |
| **Specific therapies**  *Corticosteroids*  *Antivirals (Remdesivir, Lopinavir/Ritonavir)*  *Anti-parasite (Hydroxychloroquine , Ivermectine)*  *Antibiotic (Azithromycin)*  *Anti-IL-1 (Anakinra)*  *Anti-IL-6 (Tocilizumab)* | 62%  31%  24%    14%  14%  0% | 77%  17%  0%  0%  0%  25% | 0%  0%  0%  0%  0%  0% |
| **Evolution**  *In hospital death (%)* | 10.5 | 25 | 0 |

Table S1: Demographic and clinical data of the patients.

Data are median (range) or numbers (%)

BMI: body mass index, PaO_2_: arterial oxygen pressure, FiO_2_: fraction of inspired oxygen ; NA : not applicable. * refers to statistical significance compared to M-COV ; ¶ refers to statistical significance compared to COV-ICU ; *p<0.05, **p<0.01, ***p<0.001, ****p<0.0001; ¶ p<0.05, ¶¶ p<0.01, ¶ ¶ ¶ p<0.001, ¶ ¶ ¶ ¶ p<0.0001;
