# Supplementary material for: COVID-19 PBMCs are doubly harmful, through LDN-mediated lung epithelial damage and monocytic impaired responsiveness to live Pseudomonas aeruginosa exposure
Source: Front Immunol. 2024 May 21;15:1398369. doi: 10.3389/fimmu.2024.1398369 (PMC11148249; doi:10.3389/fimmu.2024.1398369)
Supplement: Supplementary file 8 [file Table_2.docx]

Table S2 : Fluorochrome-conjugated monoclonal antibodies used in the flow cytometry panels

| Antibody | Conjugated fluorochrome | Clone | Purchase | Panel |
| --- | --- | --- | --- | --- |
| CD3 | FITC | SK7 | Invitrogen | A |
| CD8 | APC/Cyanine 7 | SK1 | Biolegend | A |
| CD4 | BV650 | RPA-T4 | BioLegend | A |
| CD33 | BV711 | P67.6 | BioLegend | A |
| CD19 | PE-Cy7 | HIB19 | BioLegend | A |
| CD11b | APC/Cyanine 7 | M1/70 | BioLegend | B |
| CD15 | BV510 | W6D3 | BioLegend | B |
| CD16 | BV711 | 3G8 | BioLegend | B |
| CD66b | PE-Cy7 | G10F5 | BioLegend | B |
| CD14 | FITC | Tuk4 | Miltenyi Biotec | B |

A: mix lymphoid

B: mix myeloid
