# Supplementary material for: COVID-19 PBMCs are doubly harmful, through LDN-mediated lung epithelial damage and monocytic impaired responsiveness to live Pseudomonas aeruginosa exposure
Source: Front Immunol. 2024 May 21;15:1398369. doi: 10.3389/fimmu.2024.1398369 (PMC11148249; doi:10.3389/fimmu.2024.1398369)
Supplement: Supplementary file 9 [file Table_3.docx]

**Lymphocytic mediators :**

Granzyme A, Granzyme B, IFN-γ, IL-13, IL-17F, IL-17 AF, CXCL-9, APRIL, IL-5, IL-2, IL-22, IL-4, CCL-5, IL-10

**Myeloid and stromal cytokines :**

C3a, CXCL5, CD14, G-CSF, sgp130 ; CXCL8, IL-1α, IL-1β, M-CSF, GM-CSF, MCP-1, MIP-2α, MIP-1α,TNF-α, eotaxin, arginase-1, IL-33, IL-12p70, IL-12p40, IL-23, lactoferrin, BAFF, S100A8/A9, TGF-α, IFN-β, IFN-α, HGF

**Proteases :**

EMMPRIN, MMP-3, MMP-1, MMP-7, MMP-12, MMP-9, MMP-2, MMP-8

Table S3
