# Supplementary material for: COVID-19 PBMCs are doubly harmful, through LDN-mediated lung epithelial damage and monocytic impaired responsiveness to live Pseudomonas aeruginosa exposure
Source: Front Immunol. 2024 May 21;15:1398369. doi: 10.3389/fimmu.2024.1398369 (PMC11148249; doi:10.3389/fimmu.2024.1398369)
Supplement: Supplementary file 10 [file Table_4.docx]

| **Organism** |  | Blood on catheter | blood | nose | Bronchopulm.  aspiration | Tracheal aspiration |  | Bronchial expectoration | BAL | throat | urine | skin | rectum |
| --- | --- | --- | --- | --- | --- | --- | --- | --- | --- | --- | --- | --- | --- |
| *Candida albicans* |  | _ | _ | _ | _ | 10% |  | 25% | _ | _ | _ | 5% | _ |
| *Serratia ureilytica* |  | _ | _ | _ | _ | _ |  | 5% | _ | _ | _ | _ | _ |
| *Serratia marcescens* |  | _ | _ | _ | _ | _ |  | 5% | _ | _ | _ | _ | _ |
| *Aspergillus niger* |  | _ | _ | _ | _ | _ |  | 5% | _ | _ | _ | _ | _ |
| *E.Coli* |  | _ | _ | 5% | _ | 5% |  | _ | _ | _ | _ | _ | 5% |
| *E. Cloacae* |  | _ | _ | _ | _ | _ |  | _ | _ | _ | _ | _ | 5% |
| *Staph.aureus* |  | _ | _ | 5% |  | _ |  | 5% | 5% | _ | _ | _ | _ |
| *Staph.epidermis* |  | _ | _ | _ | 5% | _ |  | _ | _ | _ | _ | _ | _ |
| *K.pneumoniae* |  | _ | _ | _ | 5% | _ |  | _ | _ | _ | _ | _ | _ |
| *P.aeruginosa* |  | 5% | _ | __ | 5% | _ |  | 5% | 5% | _ | _ | _ | _ |
| *Strep.pneumoniae* |  | _ | _ | _ | _ | _ |  | _ | 5% | _ | _ | _ | _ |

**Table S4 : Bacterial presence in samples (% of COV-ICU patients, n=20)**
