# Supplementary material for: COVID-19 PBMCs are doubly harmful, through LDN-mediated lung epithelial damage and monocytic impaired responsiveness to live Pseudomonas aeruginosa exposure
Source: Front Immunol. 2024 May 21;15:1398369. doi: 10.3389/fimmu.2024.1398369 (PMC11148249; doi:10.3389/fimmu.2024.1398369)
Supplement: Supplementary file 12 [file Table_6.docx]

| **Organism** |  | Presence in the respiratory tract  (nose, trachea, lung) |
| --- | --- | --- |
| *Candida albicans* |  | 25% |
| *P.aeruginosa* |  | 15% |
| *Staph aureus* |  | 15% |
| *E.Coli* |  | - |
| *E. Cloacae* |  | - |
| *Serratia marcescens* |  | 5% |
| *Aspergillus niger* |  | 5% |
| *Staph.epidermis* |  | 5% |
| *K.pneumoniae* |  | 5% |
| *Serratia ureilytica* |  | 5% |
| *Staph.pneumoniae* |  | 5% |

**Table S6 : Bacterial presence in at least one respiratory tract sample (nose, trachea, lung) (% of COV-ICU patients, n=20)**
