# Supplementary material for: COVID-19 PBMCs are doubly harmful, through LDN-mediated lung epithelial damage and monocytic impaired responsiveness to live Pseudomonas aeruginosa exposure
Source: Front Immunol. 2024 May 21;15:1398369. doi: 10.3389/fimmu.2024.1398369 (PMC11148249; doi:10.3389/fimmu.2024.1398369)
Supplement: Supplementary file 13 [file Table_7.docx]

|  | **IPF patients** |
| --- | --- |
| **Patients, n** | 17 |
| **Age, years** | 72(54-84) |
| **BMI, kg/m^2^** | 38.1 (23.9-38.1) |
| **Sex, male (%)** | 82% |
| **FVC (%)** | 89 (39-128) |
| **DLCO (%)** | 61 (21-101) |
| **CRP (mg/l)** | 5 (1-43) |
| **Oral corticoids (%)** | 0 |
| ***Antifibrotics***  *nintenanib (%)*  *pirfenidone (%)*  *nintenanib + pirfenidone (%)*  *none* | 35.2  11.7  5.8  47 |

Table S7: Demographic and clinical data of patients with idiopathic fibrosis (IPF patients).

Data are median (range) or numbers (%)

BMI: body mass index, FVC : forced vital capacity ;

DLCO : [Diffusing Capacity Of The Lungs For Carbon Monoxide](https://pubmed.ncbi.nlm.nih.gov/32310609/)

Table S7
